# Improved Automated Quantification Algorithm (AQuA) and Its Application to NMR-Based Metabolomics of EDTA-Containing Plasma

**DOI:** 10.1021/acs.analchem.0c04233

**Published:** 2021-06-15

**Authors:** Hanna E. Röhnisch, Jan Eriksson, Lan V. Tran, Elisabeth Müllner, Corine Sandström, Ali A. Moazzami

**Affiliations:** Department of Molecular Sciences, Swedish University of Agricultural Sciences, 750 07 Uppsala, Sweden

## Abstract

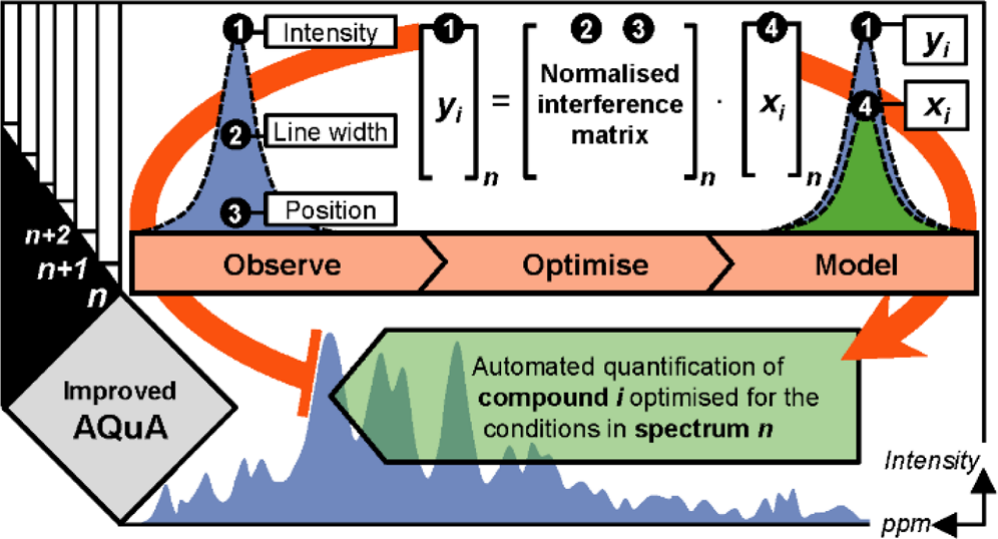

We have recently presented an Automated Quantification Algorithm (AQuA) and demonstrated its utility for rapid and accurate absolute metabolite quantification in ^1^H NMR spectra in which positions and line widths of signals were predicted from a constant metabolite spectral library. The AQuA quantifies based on one preselected signal per metabolite and employs library spectra to model interferences from other metabolite signals. However, for some types of spectra, the interspectral deviations of signal positions and line widths can be pronounced; hence, interferences cannot be modeled using a constant spectral library. We here address this issue and present an improved AQuA that handles interspectral deviations. The improved AQuA monitors and characterizes the appearance of specific signals in each spectrum and automatically adjusts the spectral library to model interferences accordingly. The performance of the improved AQuA was tested on a large data set from plasma samples collected using ethylenediaminetetraacetic acid (EDTA) as an anticoagulant (*n* = 772). These spectra provided a suitable test system for the improved AQuA since EDTA signals (i) vary in intensity, position, and line width between spectra and (ii) interfere with many signals from plasma metabolites targeted for quantification (*n* = 54). Without the improvement, ca. 20 out of the 54 metabolites would have been overestimated. This included acetylcarnitine and ornithine, which are considered particularly difficult to quantify with ^1^H NMR in EDTA-containing plasma. Furthermore, the improved AQuA performed rapidly (<10 s for all spectra). We believe that the improved AQuA provides a basis for automated quantification in other data sets where specific signals show interspectral deviations.

## Introduction

Metabolomics analyses of biofluids are widely used to study metabolic changes in relation to different pathophysiological conditions in humans.^1^ Targeted ^1^H NMR-based metabolomics can be used to quantitatively examine the metabolite content in biofluid samples.^2−4^ NMR spectra from biofluids are highly complex, and therefore quantification of metabolites requires spectral processing that can discern signals of interest and account for interference between signals originating from different compounds.^5^ It is desirable to increase the throughput of various steps in the workflow (especially in large-scale studies), and efforts have been made to automate processing systems that include, e.g., alignment of experimental spectra, identification, and/or quantification.^6−12^ In contrast to targeted methods, some quantification methods have been designed for simultaneous identification (e.g., ASICS^11^ and BAYESIL^7^). In targeted analyses, the quantification step is considered to be a major bottleneck. Therefore, there is a need for accurate and highly efficient processing of the experimental spectra.^13^

We have recently introduced an automated quantification algorithm, AQuA, that operates using spectral data extracted from a library consisting of one standard spectrum per metabolite.^14^ It was successfully implemented for rapidly quantifying metabolites in plasma samples collected using heparin as an anticoagulant. In this implementation, AQuA used one preselected NMR signal per metabolite for determining concentrations and accounted for interferences between metabolite signals, assuming that specific signal positions and line widths displayed small deviations between spectra.

Here, we introduce an improved AQuA that also includes a feature that can automatically handle interspectral deviations of signal positions and line widths for specific signals. We evaluated this new feature of AQuA with a large data set from human plasma samples that had been collected using ethylenediaminetetraacetic acid (EDTA) as an anticoagulant.^15^ EDTA prevents coagulation by binding divalent cations, and its presence in the samples yields many different NMR signals.^16,17^ These high-intensity signals interfere with signals from several plasma metabolites. Signals from free EDTA (H-EDTA^3–^) are particularly problematic due to their pH sensitivity, which can lead to interspectral deviations in signal positions and line widths.^16,18^ Spectra containing EDTA signals therefore provide an excellent test system for the improved AQuA that aims at handling such issues automatically. Using this test system and the AQuA quality indicators that we have defined and validated previously, the accuracy and speed of the improved AQuA were evaluated and compared with quantifications that did not account for variable interferences from EDTA.^14^

The improved AQuA strategy may also be used for automated quantification in high-throughput ^1^H NMR-based metabolomics of other samples that generate spectra in which positions and line widths are not stable.

## Experimental Section

In the present study, targeted ^1^H NMR-based metabolomics analyses were performed on a large set of EDTA-containing plasma samples. The analyses included sample preparation, data collection, and spectral processing as described previously.^14^ In addition, metabolite quantification was done by developing an improved version of the automated quantification algorithm (AQuA). The improvement included a feature that handles interspectral deviation of signal position and line widths. Both the improved AQuA and the AQuA without improvement were implemented and evaluated in EDTA-containing plasma.

### Sample Preparation, Collection, and Spectral Processing of ^1^H NMR Data

Human plasma samples from adult residents in Sweden have been collected and stored for future research purposes.^19^ Targeted ^1^H NMR-based metabolomics was performed on 772 plasma samples collected using EDTA as an anticoagulant. Macromolecules were removed from each plasma sample (60 μL) by ultrafiltration^14^—i.e., centrifugation (10 000*g*, 4 °C) through a 3 kDa molecular weight cut-off filter device (Amicon ultra 0.5 mL, Ultracel 3k, Merck Millipore Ltd., Tullagreen, Carrigtwohill, Co. Cork, IRL). Prior to ultrafiltration, glycerol was removed from each filter membrane by washing with water (5 mL) using centrifugation (1000*g*, 36 °C). Preparation of NMR samples: mixing each filtrate with a single solution containing H_2_O/D_2_O, phosphate buffer (pH 7.0), and trimethylsilyl-*d*_4_-propionic acid (TSP) as an internal standard, and ^1^H NMR analyses were done as previously described.^14^ All ^1^H NMR experiments were done on a Bruker III Avance spectrometer (600 MHz) with a cryogenically cooled probe. Each spectrum was recorded with 512 transients at 25 °C using the zgesgp pulse sequence (Bruker BioSpin) and the TopSpin software (version 3.1, Bruker BioSpin).

Each experimental spectrum was subjected to phase correction, adjustment of shimming irregularities (to an internal TSP signal line width of 1.10 Hz), and spectral binning (0.0002 ppm/bin) in ChenomX NMR Suite (version 7.5, ChenomX Inc., Edmonton, Canada). The binned data were imported to MATLAB (version R2012b, MathWorks Inc.) for AQuA-based processing.

### Improved AQuA Principle

As shown by Röhnisch et al.,^14^ AQuA-based processing determines the concentration of each targeted compound using the height of a preselected target signal (with relative intensity *y* and position δ_*y*_ ppm) in the experimental spectrum. This target signal is separated into (1) a reporter signal contribution (*x*) that is directly proportional to the compounds’ concentration (*c*) in the NMR sample and (2) the interference contribution (*y – x*), which is the sum of intensity contributions (≥0) from other signals located in the same spectral region. Since AQuA-based processing simultaneously considers the target signals from all targeted compounds, the output will be a set of vectors (**y̅**_**n**_, **δ̅**_**n**_, **x̅**_**n**_, and **c̅**_**n**_), where each element (*y*_*i*_, δ_*yi*_, *x*_*i*_, or *c*_*i*_) represents the value for compound *i* (*i =* 1, 2, ... *k*, if *k* different compounds are targeted) in experimental spectrum/sample *n*. Additionally, the relative interference (Δ_*i*_ elements) can be computed as (*y*_*i*_*– x*_*i*_)/*y*_*i*_ to yield a vector (Δ̅_**n**_). Each vector, **x̅**_*n*_, containing the *k* reporter signals in spectrum *n* can be computed by solving the following equation
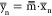
1where **y̅**_**n**_ is the *k* target signals in spectrum *n* and **m̿** is a *constantk* × *k* matrix that describes the interferences between the compounds. This interference matrix is derived from a spectral library containing one calibration spectrum for each targeted compound. Each calibration spectrum is normalized so that its reporter signal height is 1. By only considering the signal heights at the different target positions, each calibration spectrum is reduced to a calibration vector with length *k*, where each respective element represents the signal height at one of the target positions. As a result, each normalized calibration spectrum is converted to a calibration vector, where one element is 1 (i.e., the reporter signal) and the other elements are the relative signal heights (≥0) observed at the *k* – 1 remaining target positions. These normalized vectors are then organized as the columns of the interference matrix **m̿**.^14^

**Figure 1:**
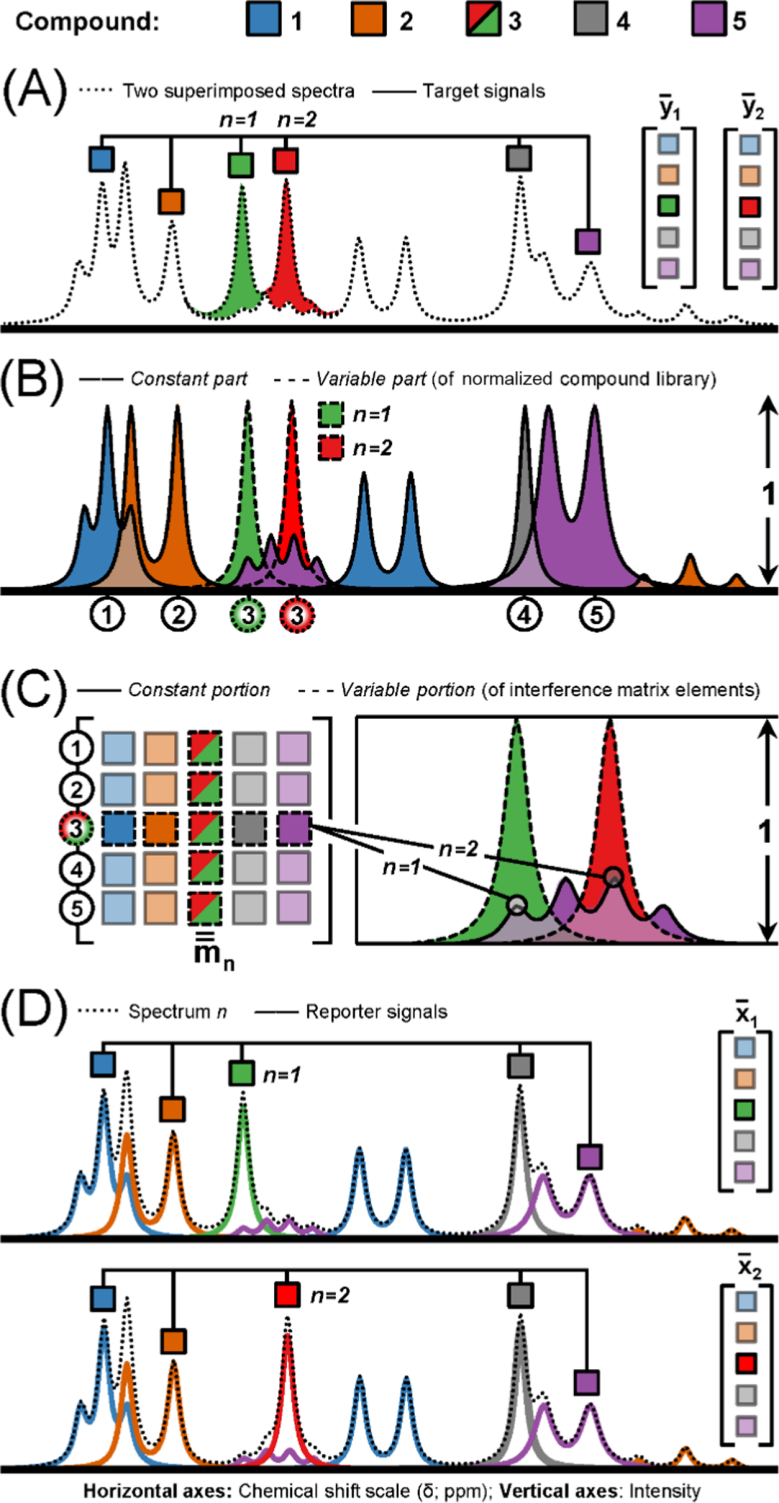
Improved AQuA principle, **y̅**_**n**_ = **m̿**_**n**_·**x̅**_**n**_, shown using a hypothetical example, where five compounds (1: blue, 2: orange, 3: green/red, 4: gray, and 5: purple) are targeted for quantification. (A) Two superimposed spectra, where the signal from compound 3 shows interspectral positional deviation (spectrum *n* = 1: green, spectrum *n* = 2: red). Target signals extracted from spectrum *n* are used in the *n*th AQuA computation (spectrum *n* = 1: **y̅**_**1**_, spectrum *n* = 2: **y̅**_**2**_). (B) Normalized compound library divided into a *constant part* that includes all compounds (1: blue, 2: orange, 4: gray, and 5: purple) with limited interspectral deviations and a *variable part* that includes the compound (3: green/red) with interspectral deviation. The *variable part* changes according to the conditions in spectrum *n*, while the *constant part* remains unchanged. (C) Interference matrix **m̿**_**n**_ optimized for spectrum *n*. The elements are divided into two portions: a *constant portion* extracted from the *constant part* of the library and a *variable portion* extracted from the *variable part* of the library. The *variable portion* changes according to the conditions in spectrum *n*, while the *constant portion* remains unchanged. (D) Results obtained with the improved AQuA (top: reporter signals **x̅**_**1**_ for spectrum *n* = 1, bottom: reporter signals **x̅**_**2**_ for spectrum *n* = 2).

We demonstrated previously that the use of a constant matrix is appropriate when signals located in the target signal regions with interference display limited interspectral positional deviation (e.g., as shown for heparin-containing plasma).^14^ In the present work, we hypothesized that if some of these signals display clear interspectral positional and/or line width deviation, the use of a constant matrix is suboptimal. We therefore introduced a strategy for improving AQuA to account for positional and line width deviations. The improved AQuA computation is done using the following equation
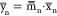
2where **y̅**_**n**_ is the *k* target signals in spectrum *n*, and where **m̿**_**n**_, is a *variablek* × *k* matrix, in which some portion of the matrix elements is changed for each spectrum *n*. In order to derive an interference matrix **m̿**_**n**_, specific for each spectrum *n*, the spectral library must to some extent be adapted to the spectrum *n*. The calibration spectrum of any given compound in the spectral library can be envisioned as a basis for identifying the signals originating from a compound in an experimental spectrum. We developed an automated peak-picking routine that can be guided to detect the signals of any given compound and determine their exact positions, line widths and heights (MATLAB script; Figure S1 in the Supporting Information). Using this information and assuming that the signals are described by Lorentzian functions, a calibration spectrum with the appropriate characteristics for the experimental spectrum *n* can be generated for any given compound. With this procedure optimized target positions and prediction of the elements in the interference matrix is obtained even if the positions and line widths of some signals vary between spectra. Hence, following the normalization procedure described above an optimized **m̿**_**n**_ matrix can be derived rapidly in each AQuA computation (Figure 1).

### AQuA Implementation

A total of 54 well-established plasma metabolites were targeted for quantification (Table S1).^14,20,21^ First, an AQuA implementation assuming only interference between metabolite signals and a constant matrix, **m̿**_constant_, for all spectra was established. Target signals were selected as previously described.^14^ Automated peak-picking (Figure S1 and Tables S2 and S3) was done to reduce each experimental spectrum *n* to a **y̅**_**n**_ vector (54 target signal elements). Data reduction and normalization of a metabolite library (spectra from 54 metabolites optimized in silico,^5^ see ref (14) for details) were done to yield a matrix **m̿**_constant_ (54 × 54), which described normalized metabolite interferences at different target positions (*m*_constant*,i,k*_∈[0,1]). The matrix **m̿**_constant_ and each **y̅**_**n**_ vector were then utilized in the AQuA computation (eq 1), thereby yielding each **x̅**_**n**_ vector (54 reporter signal elements). Each **x̅**_**n**_ vector was converted to metabolite concentrations (μM) in the NMR sample *n*.

Second, AQuA was implemented with the improved approach to also include the signals from free EDTA (H-EDTA^3–^) and two EDTA complexes (Ca–EDTA^2–^ and Mg–EDTA^2–^).^16^ The **y̅**_**n**_ vectors were thereby extended to include one target signal also for each EDTA compound, respectively. In total, each **y̅**_**n**_ vector contained 57 elements (54 from metabolites and 3 from EDTA). The portion of **m̿**_**n**_ related only to interference between the established plasma metabolites was kept constant for all spectra. Unlike most signals from plasma metabolites, signals from EDTA may vary to a significant degree in position and line width between spectra.^14,16,18^ Hence, it may be suboptimal to set the portion of the matrix elements related to the EDTA compounds constant. As part of the improved AQuA algorithm, positions and line widths (full width at half-maximum, FWHM) of signals can be derived automatically (Figure S1), and for any compound that displays clear interspectral deviation, the improved approach can be employed to optimize the portion of the matrix elements related to that compound each time the AQuA computation is performed (Figure 1; eq 2). The experimental signals from EDTA were monitored using this feature. Ca–EDTA^2–^ and Mg–EDTA^2–^ signals appeared with similar positions and line widths in all spectra, and hence, the portion of the **m̿**_**n**_ matrix that was related to Ca–EDTA^2–^ and Mg–EDTA^2–^ was kept constant for all spectra. The signals from free EDTA (H-EDTA^3–^) appeared with interspectral deviation, and therefore a new calibration spectrum *n* was created automatically using two Lorentzian functions, where positions (ppm) and line widths (Hz) matched the positions and line widths observed in spectrum *n* for free EDTA. Using this calibration spectrum (and the optimized target position for free EDTA), the portion of **m̿**_**n**_ specifically describing the interference related to free EDTA in spectrum *n* was derived automatically. Each **y̅**_**n**_ vector (57 target signal elements) and each corresponding **m̿**_**n**_ matrix (57 × 57) were used in the improved AQuA computation (eq 2). The metabolite elements in the **x̅**_**n**_ vectors (54 out of 57 elements) were converted to metabolite concentrations (μM) in the NMR sample *n*. The EDTA elements in the **x̅**_**n**_ vectors (3 out of 57 elements) were not interpreted quantitatively. Details regarding the improved AQuA implementation are shown in Figure S2 (flow chart), Table S2 (algorithm and MATLAB code), Tables S3 and S4 (input and output data) and Figure S3 (proof-of-concept figures).

## Results and Discussion

**Figure 2:**
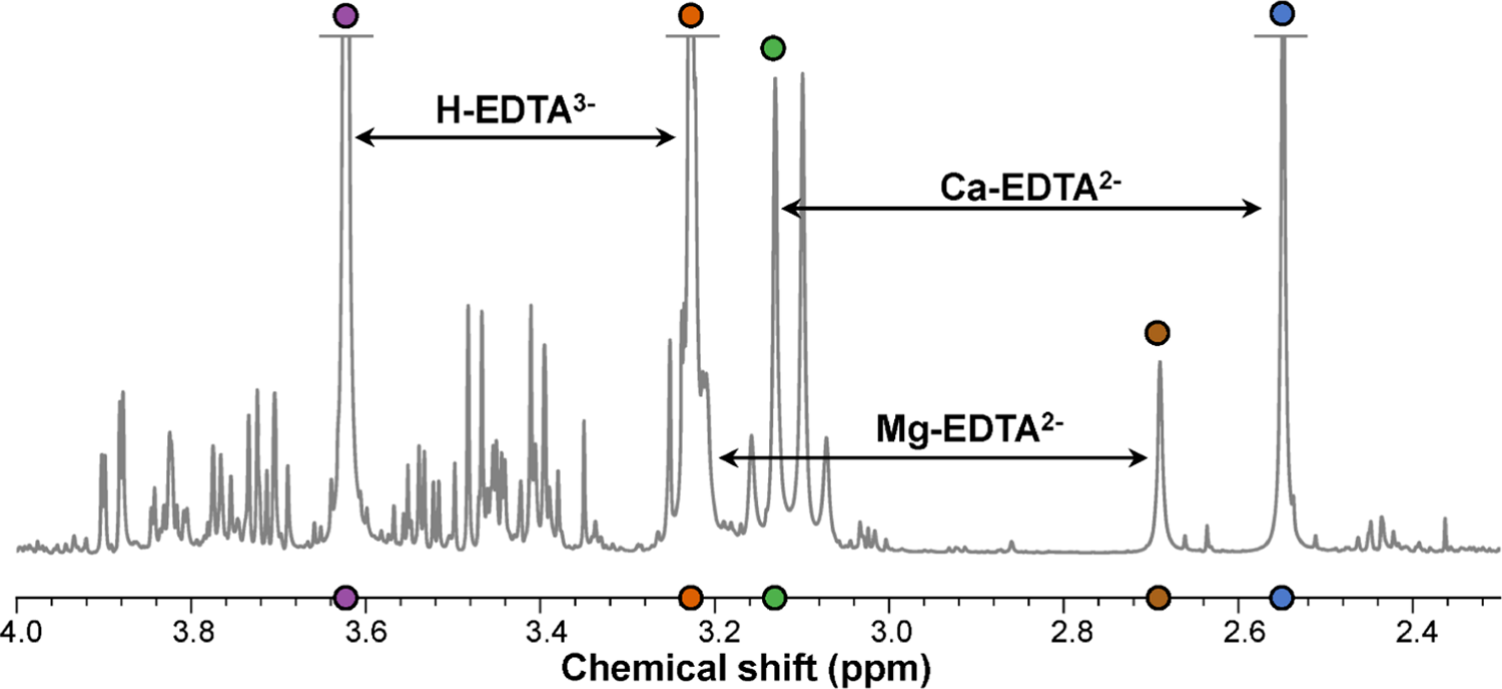
Average experimental ^1^H NMR spectrum (δ ca. 2.4–4.0 ppm) for the human plasma samples that had been collected using EDTA as an anticoagulant (*n* = 772). Purple*: H-EDTA^3–^ (δ 3.62); orange: H-EDTA^3–^ (δ 3.23); green*: Ca–EDTA^2–^ (δ 3.13); blue: Ca–EDTA^2–^ (δ 2.55); Mg–EDTA^2–^ (δ ca. 3.21; not indicated by a colored dot); and brown*: Mg–EDTA^2–^ (δ 2.69). *Selected target signals used in the improved AQuA.

Targeted ^1^H NMR-based metabolomics was applied on human plasma samples, which had been collected using EDTA as an anticoagulant. The metabolomics analyses generated a data set of 772 ^1^H NMR spectra, in which 54 human plasma metabolites were targeted for quantification with AQuA-based processing. Prior to quantification by AQuA, the ^1^H NMR signals from the anticoagulant (free EDTA as well as EDTA bound to Ca^2+^ and Mg^2+^) were investigated. The appearance (position and multiplicity) of EDTA signals in the present data set (Figure 2) was in agreement with previous observations.^16^

The EDTA signals appeared in the same regions as many of the plasma metabolite signals.^16^

**Figure 3:**
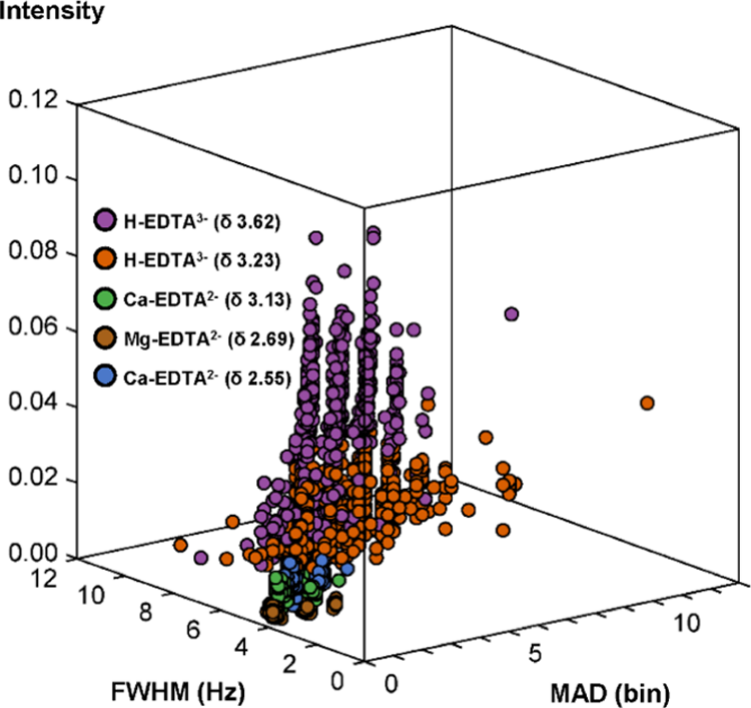
Relative intensities, positions (MAD; bin), and line widths (FWHM, Hz) for different EDTA signals in the present data set (*n* = 772). Purple: H-EDTA^3–^ (δ 3.62); orange: H-EDTA^3–^ (δ 3.23); green: Ca–EDTA^2–^ (δ 3.13); brown: Mg–EDTA^2–^ (δ 2.69); and blue: Ca–EDTA^2–^ (δ 2.55). Mg–EDTA^2–^ (δ ca. 3.21) was not evaluated due to interference with the high-intensity signal from the free EDTA signal. Details on intensities, positions, and line widths for the EDTA signals are presented in Figure S4.

Figure 3 shows the relative intensities, positions, and line widths for different EDTA signals in the data set. The line widths shown in Figure 3 are full width at half-maximum (FWHM) values. The position of each signal is shown in Figure 3 as a median absolute deviation (MAD) value—i.e., the absolute distance between the median position and the actual position, where a one-step increase in the MAD value (bin) corresponds to a positional deviation (from the median) of 0.0002 ppm. It is seen in Figure 3 that the two signals from free EDTA were generally of high intensity; however, the intensities displayed large variation in the data set. The signals from free EDTA displayed much larger deviation in line width and position compared to signals from Ca–EDTA^2–^ and Mg–EDTA^2–^ (Figure 3; for details, see Figure S4).

Hence, despite the addition of a buffer solution (during sample preparation) to minimize pH differences between samples, interspectral deviations could not be completely avoided for free EDTA signals.^16,18,22^ The MAD values of up to ca. 10 bins displayed in Figure 3 for free EDTA signals are much larger than the *positional deviations* of ca. 1 bin, which we previously reported as typical for plasma metabolites.^14^

As the free EDTA signals (Figure 2) appear close to many metabolite signals^16^ and display high intensities and large positional deviations (Figure 3) compared with typical plasma metabolite signals,^14^ this data set is challenging for metabolite quantification and therefore provides an excellent test system for demonstrating how the improved AQuA can handle interspectral deviation issues.

### Quality Indicators

In our first report on AQuA, which only accounted for interferences between metabolite signals in the absence of EDTA signals (eq 1), the results from the automated quantification were compared with a manual procedure. This comparison showed excellent agreement for most metabolites and also revealed which metabolites were difficult to quantify accurately. Furthermore, we showed that information on which metabolites are difficult to quantify could be obtained directly from values generated in the AQuA (i.e., *y*_*i*_, *δ*_*yi*_, *x*_*i*_, and Δ_*i*_) by analyzing the quality indicators *occurrence*, *positional deviation*, and degree of interference (*F_q_*).^14^ Hence, we extracted and employed these quality indicators in the present study to evaluate the improved AQuA, which also accounted for nonmetabolite signals (EDTA) as well as interspectral deviations in signal positions and line widths (eq 2).

### Occurrence

**Figure 4:**
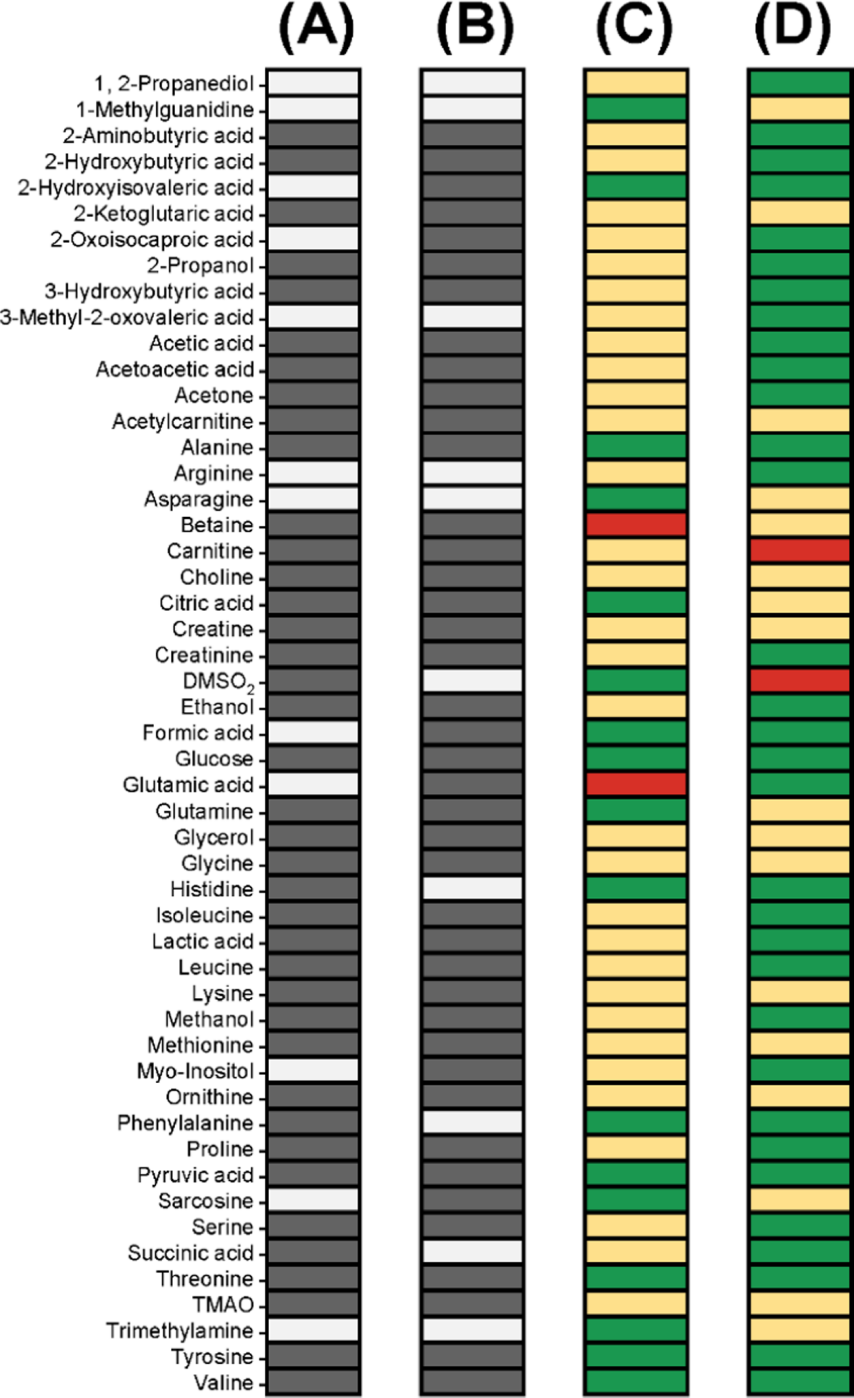
Evaluation of quality indicators in the present data set (*n* = 772) for the 51 metabolites with ≥5% *occurrence*. (A) *Occurrence* (lighter gray: 5% ≤ *occurrence* <90%, darker gray: *occurrence* ≥90%). (B) *Positional (δ)deviation* (lighter gray: *δ deviation>* 1 bin, darker gray: *δ deviation* ≤ 1 bin, 1 bin = 0.0002 ppm). (C) *F_q_* values from metabolites (red: *F0.50* > 0.50, yellow: *F0.50* ≤ 0.50 and *F0.05* > 0.00, green: *F0.05* = 0.00). (D) *F*_*q*_ values from EDTA (red: *F0.50* > 0.50, yellow: *F0.50* ≤ 0.50 and *F0.05* > 0.00, green: *F0.05* = 0.00). DMSO_2_: dimethyl sulfone, TMAO: trimethylamine-*N*-oxide. Details on quality indicators for all 54 metabolites are presented in Table S5.

The *occurrence* is the fraction of spectra in a data set for which the corresponding reporter signal occurs above the detection limit (*x*_*i*_ > 3 × noise).^14^ A too low *occurrence* disqualifies the metabolite for further quantitative and statistical analysis—i.e., the larger the *occurrence* the higher is the potential for yielding useful quantitative information. Here, the *occurrence* was computed for each metabolite (Figure 4A; see Table S5 for details). Most metabolites displayed 100% *occurrence*.

### Positional Deviation

The *positional deviation* is the value (±bins) that accounts for 95% of the median-centered distribution of target signal positions (δ_*y*_) for a given metabolite in a given data set.^14^ In agreement with our previous report, most metabolites displayed a minor (within ±1 bins) *positional deviation* (Figure 4B; see Table S5 for details). Free EDTA signals displayed much larger deviation than ±1 bins (Figure 3).

### Degree of Interference

The degree of interference is derived from the interference distribution in a data set, and *F*_*q*_ is the fraction of spectra in a data set where the interference for a given metabolite *i*, *Δ*_*i*_, exceeds a preset value *q*.^14^ The interference of each target signal can be separated into two parts: one part that originates from compounds that are metabolites and one part that originates from compounds that are nonmetabolites, such as those from EDTA (for details, see Table S6). As the interference itself can be separated, its distribution over the data set can also be separated into two parts (one distribution for other metabolites and one for nonmetabolites, respectively), whereby separate values of *F*_*q*_ can be derived.

Figure 4C shows the degree of interference from other metabolites (high: *F0.50* > 0.50, intermediate: *F0.50* ≤ 0.50 and *F0.05* > 0.00, low: *F0.05* = 0.00; see Table S5 for details). Most metabolites displayed a low or intermediate degree of interference (from other metabolite signals). AQuA quantification can tolerate a higher degree of interference provided that it occurs in a spectral region with minor *positional deviation*.^14^ It is seen in Figure 4 that the metabolites with a higher degree of interference typically displayed a low *positional deviation*. Hence, even for these metabolites, there is no severe quantification problem.

Figure 4D shows the degree of interference from EDTA (see Table S5 for details). It is seen that several metabolites displayed some degree of interference from EDTA signals. We have previously shown that AQuA may be prone to quantification errors if the degree of interference is too high (i.e., *F0.50* > 0.50).^14^ However, the degree of interference from EDTA was rarely that high, except for dimethyl sulfone (DMSO_2_) and carnitine (Figure 4D). Thus, our evaluation of the degree of interference shows that AQuA-based processing allows accurate quantification of metabolites also (i.e., acetylcarnitine and ornithine), which have previously been deemed too difficult to quantify by ^1^H NMR in plasma samples collected by using EDTA as an anticoagulant.^16^ Importantly, if these interferences from EDTA had been ignored in the implementation of AQuA, it would have resulted in inaccurate concentration estimates for these metabolites.

### Mean Sample Concentrations

**Figure 5:**
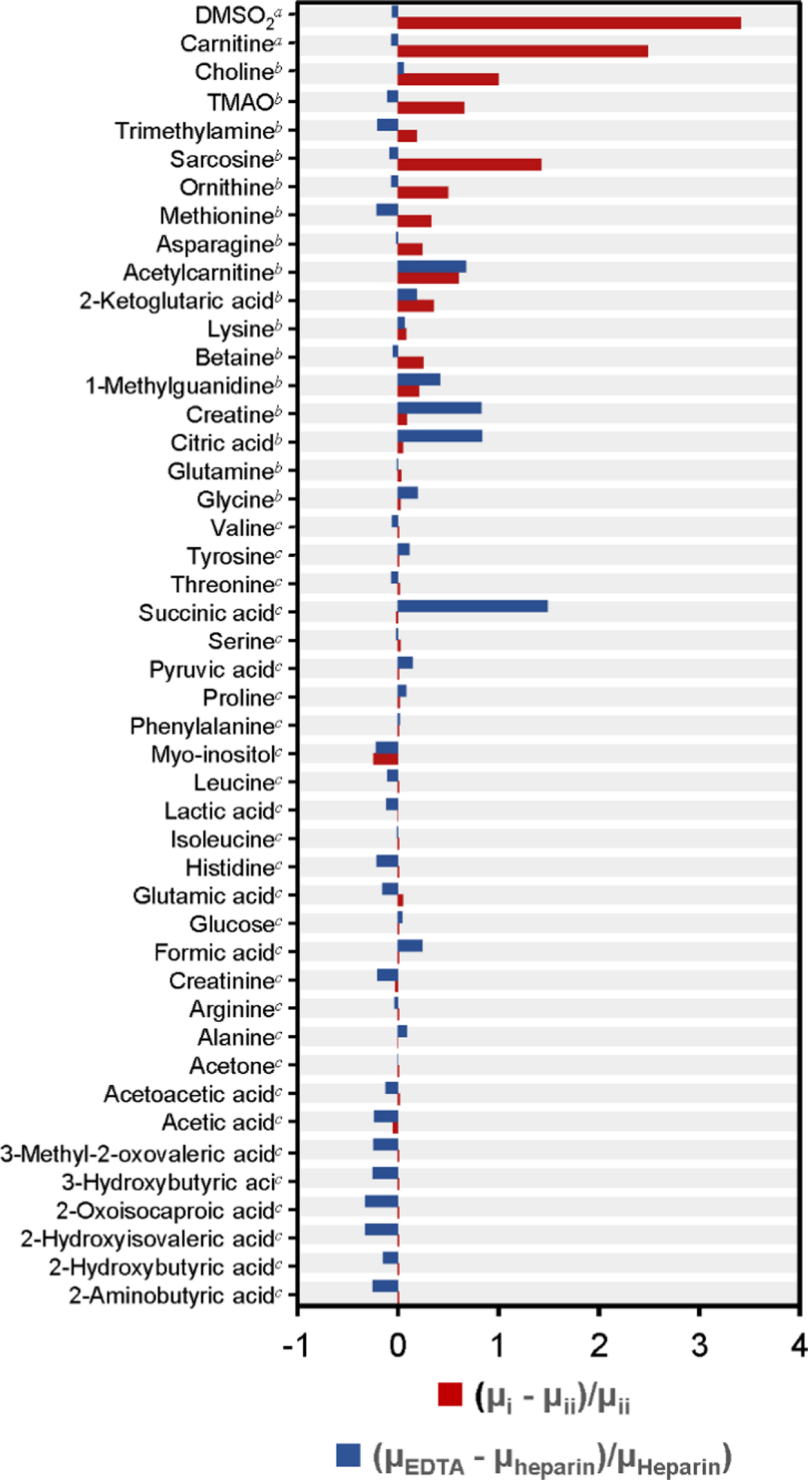
Comparison of mean metabolite concentrations (*μ*). Red bars: comparison between two different implementations of AQuA (*i*: the nonimproved AQuA, eq 1; *ii*: the improved AQuA, eq 2) employed on the EDTA data set (*n* = 772). Interference from EDTA: ^a^*F_0.50_* > 0.50, ^b^*F0.50* ≤ 0.50 and *F0.05* > 0.00, ^c^*F0.05* = 0.00. Blue bars: comparison between AQuAs optimized for the present EDTA data set and the heparin data set,^14^ respectively (*EDTA*: *n* = 772, eq 2; *heparin*: *n* = 1342, eq 1). Although included in the quantification model, alcohols are not shown since previous analyses of quality control samples revealed them to have high coefficients of variation.^14^ Details are presented in Table S7.

In the present study, the data set was analyzed with two implementations: (i) the nonimproved AQuA that accounted only for interference between metabolites (eq 1) and (ii) the improved AQuA that also accounted for interferences from free EDTA, Ca–EDTA^2–^, and Mg–EDTA^2–^ (eq 2). The magnitude of the concentration errors that would result if variable interferences from EDTA had not been accounted for was computed by comparing the resulting concentrations from the above two implementations of AQuA. The red bars in Figure 5 show the relative deviation of the mean (*μ*) concentration values for each metabolite expressed as (*μ*_*i*_ – *μ*_*ii*_)/*μ*_*ii*_, where *i* is the nonimproved AQuA and *ii* is the improved AQuA. The metabolites in Figure 5 are grouped (top-down) in descending order of *F_q_* from EDTA. The nonimproved AQuA, which did not account for interference from EDTA signals, overestimated the concentrations of ca. 20 metabolites that showed some degree of interference from EDTA (for details, see Table S7). It is seen in Figure 5 that the deviations (red bars) correlate with the size of *F*_*q*_.

We found that it could be instructive to compare the concentration errors (observed when interferences from EDTA were ignored) with some estimate of the biological variation. The results from the improved AQuA implementation for the present EDTA data set were therefore compared with concentrations derived previously for a data set from 1342 human plasma collected with heparin as an anticoagulant.^14^ The two data sets originate from two different populations (although they were generated with the same workflow for sample preparation, data collection, and spectral processing, etc.). Hence, this comparison presumably reflects some biological variations between the two datasets. For each metabolite, we computed (*μEDTA* – *μheparin*)/*μheparin*, where *EDTA* is the improved AQuA in the present EDTA data set and *heparin* is the nonimproved AQuA in the heparin data set (hence, without EDTA signals).^14^ These computed values are also displayed in Figure 5 (blue bars; see Table S7 for details). The values presented as blue bars in Figure 5 show no correlation with the size of *F*_*q*_. We note that the magnitude of concentration errors that had occurred if the variable interference from EDTA had not been accounted for in the present study (red bars in Figure 5) would have exceeded the magnitude of biological variation for many metabolites (blue bars in Figure 5). These results indicate that improving AQuA to account for the variable interferences from EDTA is required to uncover biologically meaningful metabolite data in EDTA-containing plasma.

### Efficiency

**Figure 6:**
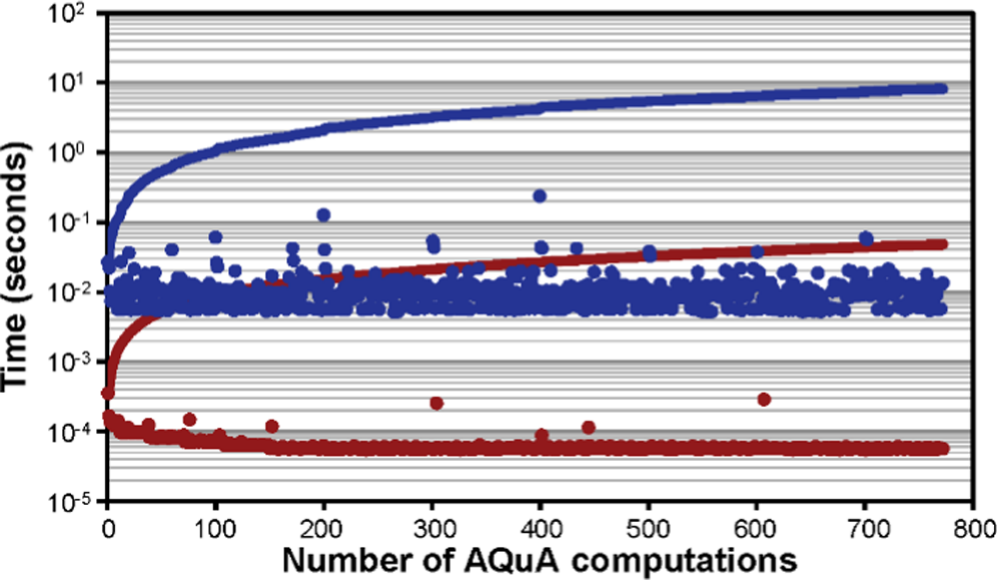
Time (seconds) required to perform the AQuA computations when quantifying 54 metabolites in the EDTA data set (*n* = 772). Blue: improved AQuA (eq 2), red: nonimproved AQuA (eq 1), dots: time per spectrum, and lines: cumulative sum of time. Peak-picking times are not included since the same strategy was used in both implementations.^14^

We have reported previously that AQuA computations are rapid.^14^ Here, we compared the time required for the nonimproved AQuA computations that employed a constant matrix **m̿** (i) and the improved AQuA computations that used a variable **m̿**_**n**_ matrix (ii). It is seen in Figure 6 that deriving a new matrix for each spectrum increases the time required for the computations. However, the improved AQuA was still very fast and required <10 s to perform all 772 computations using a standard personal computer.

### Comparison with Other Automated Quantification Approaches

The improved AQuA was also compared with ASICS,^11^ an R-based package for automated identification and quantification, which includes different approaches. For example, library signals can be aligned differently, jointly based on an entire data set or independently for each experimental spectrum. For details on different approaches tested, see Table S8. To facilitate a straightforward comparison, the ASICS workflow employed differed somewhat from the default. For example, instead of using the built-in library, we utilized the same library spectra that had been used in the improved AQuA (Table S8), and instead of applying a constant sum normalization, the normalization was based on the TSP signal (Table S8). ASICS was performed on both experimental data and simulated spectra, representing known concentrations of metabolites in mixtures (with and without EDTA signals; Table S8). The use of the simulated data revealed that it is inherently more difficult to quantify metabolites in the presence of EDTA signals, particularly due to the interspectral line width deviations of free EDTA signals (see Figure S5A,B). Furthermore, concentration estimates from the improved AQuA typically showed higher correlations with joint rather than independent ASICS procedures in experimental spectra from EDTA-containing plasma (Figure S5C,D). More details on the outcome of the ASICS computations are compiled in Table S9.

### Limitations

Compounds not included in the quantification model (e.g., potential trace contaminants^16^ or less common EDTA complexes^16^) may cause interferences that remain unaccounted for. Also, different sample preservation issues (introduced during collection or preparation) can alter the signals from, e.g., protein binding metabolites^23^ and alcohols.^3,14^ It is difficult to assess the extent of errors introduced by these uncertainties since the actual concentrations are unknown.

Furthermore, a targeted approach quantifies the same metabolites in all spectra. This can potentially be an issue since some metabolites are not detected in all spectra. However, the use of one signal for the quantification of each metabolite in AQuA results in a quantification model based on signals with the highest possible signal-to-noise ratio.^14^ Evaluation of occurrences effectively reveals remaining detection issues (Figure 4), and it is recommended to use this information (e.g., for excluding data from statistical analyses).

### Utility of the Improved AQuA

Our results show that the improved AQuA is an accurate (Figures 4 and 5) and rapid (Figure 6) processing tool desired for the many clinical and epidemiological studies that use EDTA as an anticoagulant.^16,24^ Hence, AQuA can be an attractive alternative to previous methods for analyzing EDTA-containing plasma samples that, unlike AQuA, relies on multiple NMR experiments (one-dimensional (1D) and two-dimensional (2D)) for its quantification model^25^ or reduce interferences using the J-resolved (JRES) NMR experiment^16^ (although quantification using signals from JRES spectra can be difficult^26^).

The improved AQuA may also be applicable to spectra from other types of EDTA-containing samples. For example, the use of EDTA as a chelating agent has been established in sample preparation protocols for plant extracts and urine, as chelation of dications results in interspectral stabilization of signals from some plant and urine metabolites.^27−29^ Stabilization of metabolite signals was also observed in EDTA-containing plasma—e.g., histidine showed smaller positional deviation here (Table S5) compared to our previous study.^14^ Therefore, spectra from EDTA-containing plant and urine samples can be potential areas of application for the improved AQuA. Naturally, spectral libraries specific for plant extracts or urine must be developed and employed together with the strategy for improving AQuA.

Although the improved AQuA was demonstrated using EDTA-containing plasma as a test system, the underlying strategy may be applied to other (metabolite or nonmetabolite) compounds that display similar issues as free EDTA showed in the present data set: (1) the compounds’ signals are located in complex regions of the spectrum where many other compounds display signals (Figure 2) and (2) the compounds’ signals display positional and/or line width deviations between spectra (Figure 3). According to the improved AQuA strategy, such compounds are recognized by automated determination of their signal positions and line widths in the data set, and this information is utilized to vary the elements of the interference matrix accordingly in the computations (eq 2). Interferences from such compounds are thereby accurately accounted for despite interspectral deviation issues (Figure 1). Hence, this strategy increases the flexibility of AQuA and facilitates the processing of data sets with more complex signal patterns.

### Outlooks

It is desirable to increase the efficiency of the entire workflow (not only the final quantification step). However, the outcome of the quantification can vary depending on how data are generated. For example, removal of macromolecules during sample preparation or filtering out their signals with the Carr–Purcell–Meiboom–Gill experiment yield somewhat different spectra.^23^ Additional studies are required to assess the most time/cost beneficial way to generate data with a quality suitable for quantification. Automated systems for processing generated data have been presented based on other quantification models.^6,7,11^ In the future, it could be possible to develop a completely automated system for high-throughput data processing, where all prior steps are streamlined for targeted quantification with the rapid improved AQuA strategy.

## Conclusions

The improved AQuA provides a means for handling interferences despite varying positions and line widths between spectra for specific signals. The algorithm performance was demonstrated using a large set of ^1^H NMR spectra from human plasma collected using EDTA as an anticoagulant (*n* = 772). Signals from EDTA vary in intensity, position, and line width between spectra and interfere with signals from known human plasma metabolites. The improved AQuA handled these interferences and allowed quantification even of metabolites such as acetylcarnitine and ornithine that display severe interference from EDTA signals. The improved AQuA implementation is suitable for high-throughput applications since it required <10 s for the quantification of 54 plasma metabolites in 772 spectra. We believe that the improved AQuA is a desired processing tool for NMR data from clinical and epidemiological studies that collects plasma with EDTA as an anticoagulant. Beyond this, the improved AQuA provides a basis for automated quantification in other types of samples that may generate NMR spectra in which specific signal positions and line widths are not stable between spectra.

## Supporting Information Available

Algorithm for automatically determining height, position, and line width (Figure S1); metabolites targeted for quantification (Table S1); improved AQuA implementation for EDTA-containing plasma (Figures S2 and S3, and Tables S2–S4); intensities, positions, and line widths for different EDTA signals (Figure S4); quality indicators for metabolites quantified with the improved AQuA (Table S5); separating the interference into different sources (Table S6); comparisons of mean sample concentrations (Table S7); and comparisons with ASICS (Tables S8, S9, and Figure S5) (PDF)
