# Supplementary material for: Improved Automated Quantification Algorithm (AQuA) and Its Application to NMR-Based Metabolomics of EDTA-Containing Plasma
Source: Anal Chem. 2021 Jun 15;93(25):8729–38. doi: 10.1021/acs.analchem.0c04233 (PMC8253485; doi:10.1021/acs.analchem.0c04233)
Supplement: Supplementary file 1 — ac0c04233_si_001.pdf [file ac0c04233_si_001.pdf]

Supporting Information

An Improved Automated Quantification Algorithm (AQuA) and Its Application to NMR-based Metabolomics of EDTA-Containing Plasma

Hanna E. Röhnisch[1], Jan Eriksson[1], Lan V. Tran[1], Elisabeth Müllner, Corine Sandström[1], Ali A. Moazzami[1]

[1] *Department of Molecular Sciences, Swedish University of Agricultural Sciences, Uppsala, Sweden*

Table of content

Figure S1: Signal monitoring algorithm for automatically determining height, position and line width

Table S1: Metabolites targeted for quantification

Figure S2: Flowchart for the improved AQuA

Table S2: Demonstrating the implementation of the improved AQuA for EDTA-containing plasma

Table S3: Explanation of in- and outputs for the improved AQuA implementation

Table S4: Additional information data used for the improved AQuA implementation

Figure S3: Proof-of concept figures for the improved AQuA implemented in MATLAB

Figure S4: Intensities, positions and line widths for different EDTA signals

Table S5: Quality indicators for metabolites quantified with the improved AQuA

Table S6: Separating the interference into different sources

Table S7: Comparison of mean sample concentrations

Table S8: Workflow for comparing the improved AQuA to different ASICS approaches

Figure S5: Correlation heatmap for 54 metabolites

Table S9: General observations and interpretations of comparisons with ASICS

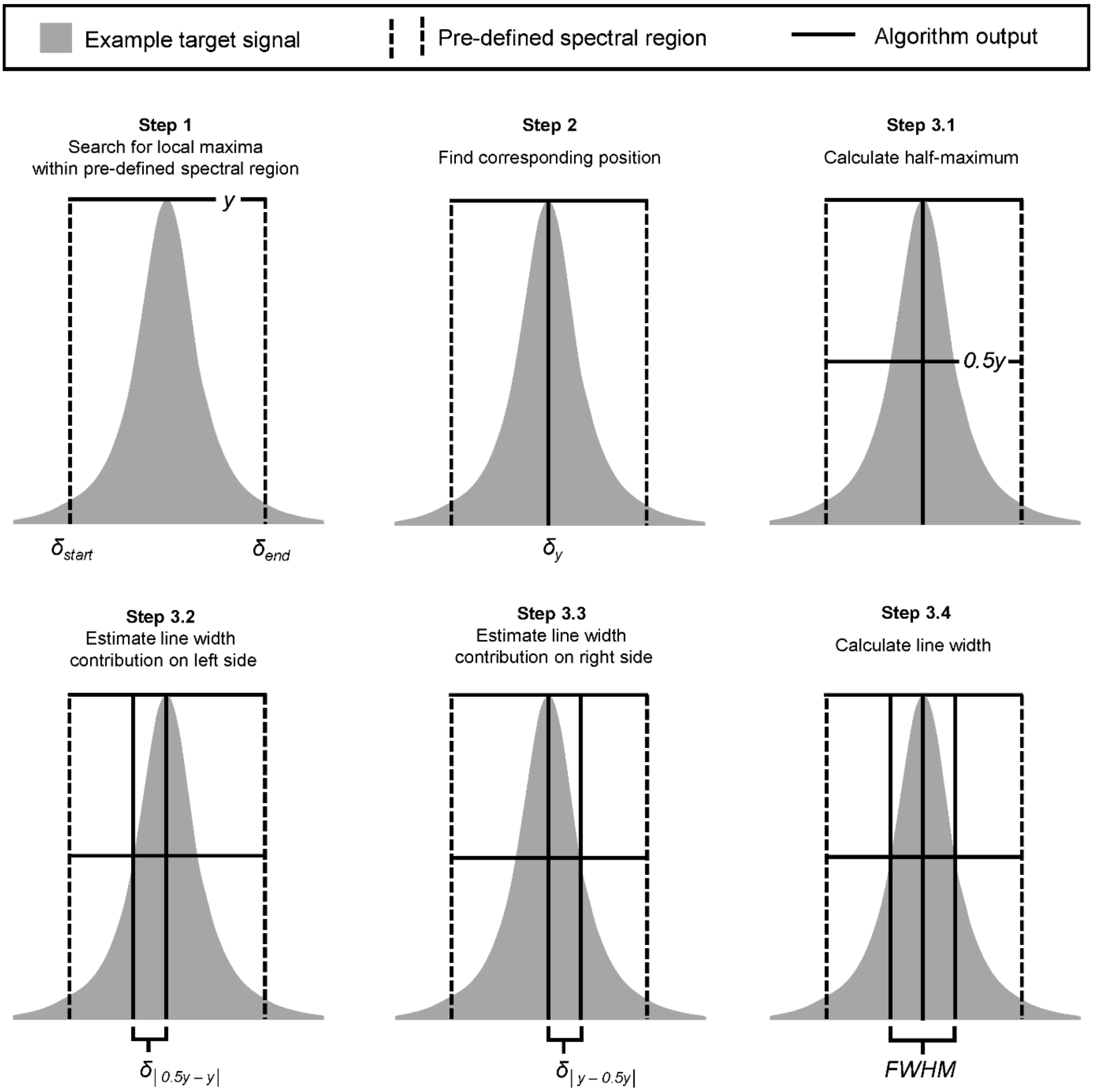

Figure S1. Signal monitoring algorithm for automatically determining height (i.u.), position (ppm) and line width (ppm). Grey: Example target signal in an experimental spectrum. Black dashed line: Pre-selected spectral region where the signal is expected (guided by data in the metabolite library). Black line: Algorithm outputs. Step 1: Determine the height (y, i.u.) by searching for the local maximum within the pre-selected spectral region (δ_{start} to δ_{end}, ppm). Step 2: Find the corresponding position (δ_y, ppm). Step 3.1: Calculate the half-height ($0.5y$, i.u.). Step 3.2: Estimate the line width contribution (ppm) on the left side of the signal ($\delta_{|0.5y-y|}$; distance between position δ_y and, to the left, the position that corresponds to the intensity closest to $0.5y$). Step 3.3: Estimate the line width contribution (ppm) on the right side of the signal ($\delta_{|y-0.5y|}$; distance between position δ_y and, to the right, the position that corresponds to the intensity closest to $0.5y$). Step 3.4: Calculate the line width ($FWHM$, ppm) as the sum of contributions ($\delta_{|0.5y-y|} + \delta_{|y-0.5y|}$). For details on how to implement the code in MATLAB, see Table S2. **Abbreviations:** FWHM, full width at half-maximum; i.u. intensity units; ppm, parts per million.

Table S1: Metabolites targeted for quantification with AQuA [a, b]

Metabolite	HMDB ID	InChI
1-Methylguanidine	0001522	InChI=1S/C2H7N3/c1-5-2(3)4/h1H3,(H4,3,4,5)
1, 2-Propanediol	0001881	InChI=1S/C3H8O2/c1-3(5)2-4/h3-5H,2H2,1H3/t3-/m1/s1
2-Aminobutyric acid	0000452	InChI=1S/C4H9NO2/c1-2-3(5)4(6)7/h3H,2,5H2,1H3,(H,6,7)/t3-/m0/s1
2-Hydroxybutyric acid	0000008	InChI=1S/C4H8O3/c1-2-3(5)4(6)7/h3,5H,2H2,1H3,(H,6,7)/t3-/m0/s1
2-Hydroxyisovaleric acid	0000407	InChI=1S/C5H10O3/c1-3(2)4(6)5(7)8/h3-4,6H,1-2H3,(H,7,8)
2-Ketoglutaric acid	0000208	InChI=1S/C5H6O5/c6-3(5(9)10)1-2-4(7)8/h1-2H2,(H,7,8)(H,9,10)
2-Oxoisocaproic acid	0000695	InChI=1S/C6H10O3/c1-4(2)3-5(7)6(8)9/h4H,3H2,1-2H3,(H,8,9)
2-Propanol	0000863	InChI=1S/C3H8O/c1-3(2)4/h3-4H,1-2H3
3-Hydroxybutyric acid	0000011	InChI=1S/C4H8O3/c1-3(5)2-4(6)7/h3,5H,2H2,1H3,(H,6,7)/t3-/m1/s1
3-Methyl-2-oxovaleric acid	0000491	InChI=1S/C6H10O3/c1-3-4(2)5(7)6(8)9/h4H,3H2,1-2H3,(H,8,9)
Acetic acid	0000042	InChI=1S/C2H4O2/c1-2(3)4/h1H3,(H,3,4)
Acetoacetic acid	0000060	InChI=1S/C4H6O3/c1-3(5)2-4(6)7/h2H2,1H3,(H,6,7)
Acetone	0001659	InChI=1S/C3H6O/c1-3(2)4/h1-2H3
Acetylcarnitine	0000201	InChI=1S/C9H17NO4/c1-7(11)14-8(5-9(12)13)6-10(2,3)4/h8H,5-6H2,1-4H3/t8-/m1/s1
Alanine	0000161	InChI=1S/C3H7NO2/c1-2(4)3(5)6/h2H,4H2,1H3,(H,5,6)/t2-/m0/s1
Arginine	0000517	InChI=1S/C6H14N4O2/c7-4(5(11)12)2-1-3-10-6(8)9/h4H,1-3,7H2,(H,11,12)(H4,8,9,10)/t4-/m0/s1
Asparagine	0000168	InChI=1S/C4H8N2O3/c5-2(4(8)9)1-3(6)7/h2H,1,5H2,(H2,6,7)(H,8,9)/t2-/m0/s1
Betaine	0000043	InChI=1S/C5H11NO2/c1-6(2,3)4-5(7)8/h4H2,1-3H3
Carnitine	0000062	InChI=1S/C7H15NO3/c1-8(2,3)5-6(9)4-7(10)11/h6,9H,4-5H2,1-3H3/t6-/m1/s1
Choline	0000097	InChI=1S/C5H14NO/c1-6(2,3)4-5-7/h7H,4-5H2,1-3H3/q+1
Citric acid	0000094	InChI=1S/C6H8O7/c7-3(8)1-6(13,5(11)12)2-4(9)10/h13H,1-2H2,(H,7,8)(H,9,10)(H,11,12)
Creatine	0000064	InChI=1S/C4H9N3O2/c1-7(4(5)6)2-3(8)9/h2H2,1H3,(H3,5,6)(H,8,9)
Creatinine	0000562	InChI=1S/C4H7N3O/c1-7-2-3(8)6-4(7)5/h2H2,1H3,(H2,5,6,8)
$DMSO_2$	0004983	InChI=1S/C2H6O2S/c1-5(2,3)4/h1-2H3
Ethanol	0000108	InChI=1S/C2H6O/c1-2-3/h3H,2H2,1H3
Formic acid	0000142	InChI=1S/CH2O2/c2-1-3/h1H,(H,2,3)
Glucose	0000122	InChI=1S/C6H12O6/c7-1-2-3(8)4(9)5(10)6(11)12-2/h2-11H,1H2/t2-,3-,4+,5-,6-/m1/s1
Glutamic acid	0000148	InChI=1S/C5H9NO4/c6-3(5(9)10)1-2-4(7)8/h3H,1-2,6H2,(H,7,8)(H,9,10)/t3-/m0/s1
Glutamine	0000641	InChI=1S/C5H10N2O3/c6-3(5(9)10)1-2-4(7)8/h3H,1-2,6H2,(H2,7,8)(H,9,10)/t3-/m0/s1

Table S1: (Continued)

Metabolite	HMDB ID	InChI
Glycerol	0000131	InChI=1S/C3H8O3/c4-1-3(6)2-5/h3-6H,1-2H2
Glycine	0000123	InChI=1S/C2H5NO2/c3-1-2(4)5/h1,3H2,(H,4,5)
Hippuric acid	0000714	InChI=1S/C9H9NO3/c11-8(12)6-10-9(13)7-4-2-1-3-5-7/h1-5H,6H2,(H,10,13)(H,11,12)
Histidine	0000177	InChI=1S/C6H9N3O2/c7-5(6(10)11)1-4-2-8-3-9-4/h2-3,5H,1,7H2,(H,8,9)(H,10,11)/t5-/m0/s1
Isoleucine	0000172	InChI=1S/C6H13NO2/c1-3-4(2)5(7)6(8)9/h4-5H,3,7H2,1-2H3,(H,8,9)/t4-,5-/m0/s1
Lactic acid	0000190	InChI=1S/C3H6O3/c1-2(4)3(5)6/h2,4H,1H3,(H,5,6)/t2-/m0/s1
Leucine	0000687	InChI=1S/C6H13NO2/c1-4(2)3-5(7)6(8)9/h4-5H,3,7H2,1-2H3,(H,8,9)/t5-/m0/s1
Lysine	0000182	InChI=1S/C6H14N2O2/c7-4-2-1-3-5(8)6(9)10/h5H,1-4,7-8H2,(H,9,10)/t5-/m0/s1
Methanol	0001875	InChI=1S/CH4O/c1-2/h2H,1H3
Methionine	0000696	InChI=1S/C5H11NO2S/c1-9-3-2-4(6)5(7)8/h4H,2-3,6H2,1H3,(H,7,8)/t4-/m0/s1
Myo-Inositol	0000211	InChI=1S/C6H12O6/c7-1-2(8)4(10)6(12)5(11)3(1)9/h1-12H
Ornithine	0000214	InChI=1S/C5H12N2O2/c6-3-1-2-4(7)5(8)9/h4H,1-3,6-7H2,(H,8,9)/t4-/m0/s1
Phenylalanine	0000159	InChI=1S/C9H11NO2/c10-8(9(11)12)6-7-4-2-1-3-5-7/h1-5,8H,6,10H2,(H,11,12)/t8-/m0/s1
Proline	0000162	InChI=1S/C5H9NO2/c7-5(8)4-2-1-3-6-4/h4,6H,1-3H2,(H,7,8)/t4-/m0/s1
Propionic acid	0000237	InChI=1S/C3H6O2/c1-2-3(4)5/h2H2,1H3,(H,4,5)
Pyruvic acid	0000243	InChI=1S/C3H4O3/c1-2(4)3(5)6/h1H3,(H,5,6)
Sarcosine	0000271	InChI=1S/C3H7NO2/c1-4-2-3(5)6/h4H,2H2,1H3,(H,5,6)
Serine	0000187	InChI=1S/C3H7NO3/c4-2(1-5)3(6)7/h2,5H,1,4H2,(H,6,7)/t2-/m0/s1
Succinic acid	0000254	InChI=1S/C4H6O4/c5-3(6)1-2-4(7)8/h1-2H2,(H,5,6)(H,7,8)
Threonine	0000167	InChI=1S/C4H9NO3/c1-2(6)3(5)4(7)8/h2-3,6H,5H2,1H3,(H,7,8)/t2-,3+/m1/s1
Trigonelline	0000875	InChI=1S/C7H7NO2/c1-8-4-2-3-6(5-8)7(9)10/h2-5H,1H3
Trimethylamine	0000906	InChI=1S/C3H9N/c1-4(2)3/h1-3H3
TMAO	0000925	InChI=1S/C3H9NO/c1-4(2,3)5/h1-3H3
Tyrosine	0000158	InChI=1S/C9H11NO3/c10-8(9(12)13)5-6-1-3-7(11)4-2-6/h1-4,8,11H,5,10H2,(H,12,13)/t8-/m0/s1
Valine	0000883	InChI=1S/C5H11NO2/c1-3(2)4(6)5(7)8/h3-4H,6H2,1-2H3,(H,7,8)/t4-/m0/s1

[a] **HMDB IDs** were obtained from the Human Metabolome Database (https://hmdb.ca/ Accessed on May 18, 2020)

[b] **InChIs** were obtained from the National Center for Biotechnology Information, PubChem Database (https://pubchem.ncbi.nlm.nih.gov/ Accessed on May 18, 2020)

Abbreviations: $DMSO_2$, dimethyl sulfone; TMAO, trimethylamine-N-oxide

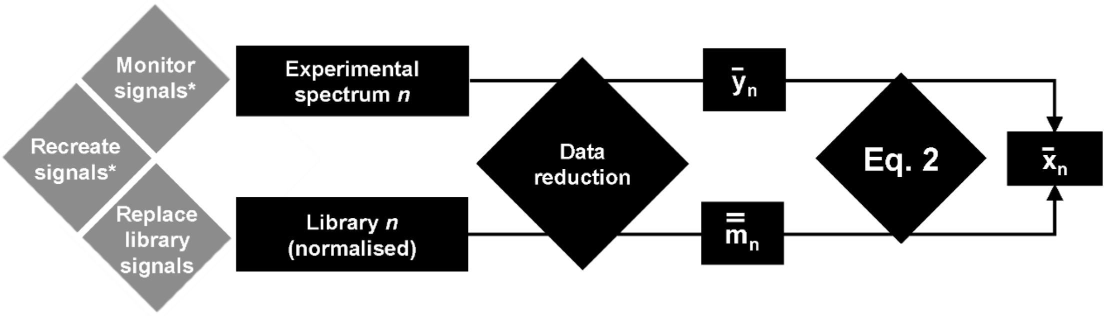

Figure S2. Flowchart for the improved AQuA. Black: features that can be implemented using the same methodology used in the non-improved AQuA (for details, see Röhnisch, H. E.; Eriksson, J.; Müllner, E.; Agback, P.; Sandström, C.; Moazzami, A. A. *Analytical Chemistry* **2018**, *90*, 2095-2102). For example, the process of generating $\bar{\bar{\mathbf{m}}}_{\mathbf{n}}$ from library *n* remains identical as the generation of $\bar{\bar{\mathbf{m}}}$ from the fixed library, and in that follows that eq. 1 ($\bar{\mathbf{y}}_{\mathbf{n}} = \bar{\bar{\mathbf{m}}} \cdot \bar{\mathbf{x}}_{\mathbf{n}}$) becomes eq. 2 ($\bar{\mathbf{y}}_{\mathbf{n}} = \bar{\bar{\mathbf{m}}}_{\mathbf{n}} \cdot \bar{\mathbf{x}}_{\mathbf{n}}$). Grey: The new feature of the improved AQuA is the automated generation of library *n*. In this study, the new feature was employed on free EDTA* since its signals showed inter-spectral deviation issues (Figure 3). **Monitor signals:** EDTA is detected as two singlets (Figure 2). The intensity, position and line width of each singlet is monitored in experimental spectrum *n*. **Recreate signals:** The output from the monitoring process of each free EDTA singlet (and the chemical shift scale) is used as input in the Lorentzian function to recreate each signal. **Replace fixed library:** The corresponding free EDTA signals in the fixed compound library are replaced with the recreated library signals for free EDTA so that data reduction and the AQuA computation (eq 2) can be done to generate $\bar{\bar{\mathbf{m}}}_{\mathbf{n}}$.

Table S2: Demonstrating the implementation of the improved AQuA for EDTA-containing plasma [a]

Algorithm Normalise the fixed library and generate calibration factors

Input lib_spectra(1:42000 × 1:57); lib_window(1:57 × 1:2); lib_conc_um(1:57 × 1)

Code

```
for i=1:57;
[reporter_height(i,1), target_position(i,1)] = max(lib_spectra(lib_window(i,1):lib_window(i,2),i));
                       target_position(i,1)  = (lib_window(i,1)-1) + target_position(i,1);
normalised_lib_spectra(1:42000,i)            = lib_spectra(1:42000,i)/reporter_height(i,1);
calibration_factor(i,1)                      = reporter_height(i,1)/lib_conc_um(i,1);
end;
```

Output normalised_lib_spectra(1:42000 × 1:57); calibration_factor(1:57 × 1); target_position(1:57 × 1);

Algorithm Data reduction of experimental spectrum *n* by automated peak-picking to generate the target signals ($\bar{\mathbf{y}}_\mathbf{n}$)

Input Experimental spectrum *n*: exp_spectra(1:42000, n); optimised peak-picking windows: exp_window(1:57 × 2)

Code*

```
n=input('experimental spectrum nr: ');
for i=1:57;
[yn(i,n), yn_position(i,n)] = max(exp_spectra(exp_window(i,1):exp_window(i,2),n));
          yn_position(i,n)  = (exp_window(i,1)-1) + yn_position(i,n);
end;
```

Output yn (1:57, n); yn_position(1:57, n)

Algorithm Signal monitoring of free EDTA* in experimental spectrum *n*

Input exp_spectra(1:42000, n)

Code

```
a1=21360; b1=21415; c1=50; % 1st signal (free EDTA at ca. 3.62 ppm)
[S1(n,1), S1(n,2)] = max(exp_spectra(a1:b1,n));
          S1(n,2)  = (a1-1) + S1(n,2);
[S1(n,3), S1(n,4)] = min(abs(exp_spectra((S1(n,2)-c1):S1(n,2),n)-0.5*S1(n,1)));
          S1(n,4)  = (S1(n,2)-c1-1) + S1(n,4);
[S1(n,5), S1(n,6)] = min(abs(exp_spectra(S1(n,2):((S1(n,2)+c1)),n)-0.5*S1(n,1)));
          S1(n,6)  = (S1(n,2)-1) + S1(n,6);
          S1(n,7)  = (abs(S1(n,4)-S1(n,6)))*0.0002;
a2=23350; b2=23374; c2=35; % 2nd signal (free EDTA at ca. 3.23 ppm)
[S2(n,1), S2(n,2)] = max(exp_spectra(a2:b2,n));
          S2(n,2)  = (a2-1) + S2(n,2);
[S2(n,3), S2(n,4)] = min(abs(exp_spectra((S2(n,2)-c2):S2(n,2),n)-0.5*S2(n,1)));
          S2(n,4)  = (S2(n,2)-c2-1) + S2(n,4);
          S2(n,5)  = (abs(S2(n,2)-S2(n,4)));
          S2(n,6)  = S2(n,5)*2*0.0002;
```

Output S1(n, 1:7); S2(n, 1:6)

Algorithm Recreation of library signals for free EDTA* for computation *n*

Input Lorentzian.m; position, S2(n, 2); S1(n, 7); S2(n, 2); S2(n, 6)

Code

```
lib_S1(:,n)     = Lorentzian(ppm, (ppm(S1(n,2))-0.0001), S1(n,7));
lib_maxS1(n,1) = max(lib_S1(:,n));
lib_S1(:,n)     = lib_S1(:,n)/lib_maxS1(n,1);
lib_S2(:,n)     = Lorentzian(ppm, (ppm(S2(n,2))-0.0001), S2(n,6));
lib_maxS2(n,1) = max(lib_S2(:,n));
lib_S2(:,n)     = lib_S2(:,n)/lib_maxS2(n,1);
lib_HEDTA(:,n) = lib_S1(:,n) + (lib_S2(:,n)*0.5181);
```

Output lib_HEDTA(1:42000, n)

Algorithm Generate normalized library *n* by replacing the fixed spectrum for free EDTA* with the recreated spectrum from (8).

Input normalised_lib_spectra(1:42000 × 1:57); lib_HEDTA(1:42000, n)

Code

```
normalised_lib_spectra_n              = normalised_lib_spectra;
normalised_lib_spectra_n(1:42000,21) = lib_HEDTA(:,n);
```

Output normalised_lib_spectra_n(1:42000 × 1:57)

Algorithm Generate interference matrix $\bar{\bar{\mathbf{m}}}_\mathbf{n}$ for computation *n*

Input normalised_lib_spectra_n(1:42000 × 57); target_position(1:57 × 1)

Code

```
for i=1:57;
target_position_n(i,n) = target_position(i,1);
target_position_n(21,n)= yn_position(21,n);
mn(i,:) = normalised_lib_spectra_n(target_position_n(i,n),:);
end;
```

Output mn(1:57 × 1:57)

Algorithm Derive reporter signals ($\bar{\mathbf{x}}_\mathbf{n}$) with eq 2

Input mn(1:57 × 1:57); yn (1:57, n)

Code

```
xn(:,n) = mn\yn(:,n);
```

Output xn (1:57, n)

Algorithm Absolute quantitation in NMR sample *n* (µM, 1 decimal)

Input xn (1:57, n); calibration_factor(1:57,1)

Code

```
c_sample_um(:,n) = xn(:,n)./calibration_factor;
c_sample_um(:,n) = round(c_sample_um(:,n)*10)/10;
```

Output c_sample_uM(1:57, n)

Algorithm (for proof-of-concept if desired)

Input exp_spectra(1:42000); yn(1:57, n); xn(1:57,n); normalised_lib_spectra_n(1:42000 × 1:57); ppm(1:42000;1)

Code

```
for i=1:57;
library_xn(:,i) = normalised_lib_spectra_n(:,i).*xn(i,n);
end;
for i=1:42000;
library_xn_sum(i,n)=sum(library_xn(i,:));
end;
figure; set(gca,'xdir','reverse'); hold on;
plot(ppm, exp_spectra(:,n), 'k');
plot(ppm(yn_position(:,n)), yn(:,n),'.k');
plot(ppm, library_xn_sum(:,n), 'b');
plot(ppm, library_xn, 'r');
plot(ppm(target_position_n(:,n)), xn(:,n),'.r'); hold off;
```

Output library_xn (1:42000 × 1:57); library_xn_sum(1:42000, n)

---

[a] In order to e.g., perform the peak-picking in all experimental spectra, do a for-loop in that code section (for n=1:772; [code*] end;) Peak-picking of target signals in all 772 experimental spectra (measured with tic; toc;) was done in ca. 0.13 seconds

Table S3: Explanation of in- and outputs for the improved AQuA implementation [a,b,c]

Variables	Content
**Input**	
exp_spectra(1:42000 × 1:772)	Binned data for each of the 772 experimental spectra from the EDTA-containing plasma samples
exp_name_list(1:772 × 1)	The name of each experimental spectrum
ppm(1:42000 × 1)	The recreated ppm scale
exp_window(1:57 × 1:2)	Windows for automated peak-picking used in data reduction of the experimental spectra (the values are presented in Table S4)
lib_spectra(1:42000 × 1:57)	Binned data for each of compounds in the (fixed) compound library
lib_name_list(1:57 × 1)	The name of each compound in the (fixed) library (the names are presented in Table S4)
lib_conc_um(1:57 × 1)	The concentration of each compound in the (fixed) library (Table S4).
lib_window(1:57 × 1:2)	Windows for automated peak-picking used in data reduction of the compound library (the values are presented in Table S4)
Lorentzian.m	`function [HEDTA]= Lorentzian(ppm, position, width)`<br>`HEDTA=(((1/pi)*(0.5*width)./(((ppm-(position)).^2)+(0.5*width)^2)));`
a1; b1; c1	Parameters used in the signal monitoring of the first free EDTA signals (S1)
a2; b2; c2	Parameters used in the signal monitoring of the second free EDTA signals (S2)
**Output**	
reporter_height(1:57 × 1)	Height of all reporter signals in the (fixed) compound library
target_position(1:57 × 1)	Position of all reporter signals in the (fixed) compound library
calibration_factor(1:57, 1)	The ratio between metabolite concentrations and reporter heights in the (fixed) compound library
normalised_lib_spectra(1:42000 × 1:57)	The normalised (fixed) compound library (one spectrum per compound)
yn(1:57, n)	Target signal heights in experimental spectrum *n*
yn_position(1:57, n)	Corresponding positions in experimental spectrum *n*. To convert to ppm do ppm(target_position);
S1(n, 1:7)	Outcome from signal monitoring of the first free EDTA signal (target signal) in experimental spectrum *n*:1, height; 2, position (bin); 7, line width (ppm). To convert to Hertz, multiply with spectrometer frequency (600 MHz)
S2(n, 1:6)	Outcome from signal monitoring of second free EDTA signal in experimental spectrum *n*: 1, height; 2, position (bin); 6, line width (ppm). To convert to Hertz, multiply with spectrometer frequency (600 MHz)
lib_HEDTA(1:42000, n)	Normalised library spectrum *n* for free EDTA, matched based on its conditions (positions and line widths) in experimental spectrum *n*. The ratio between the two signals in the library was set to a fixed value in all 772 library spectra (i.e., the slope in the regression line between the intensities of the two experimental signals from free EDTA in the entire dataset; n=772; k=0.5181; r^2=0.9976).
normalised_lib_spectra_n(1:42000 × 1:57)	Normalised library *n* where the fixed spectrum for free EDTA (normalised) has been replaced with normalised library spectrum *n* for free EDTA
target_position_n(1:57 × 1)	Same as target_position, except that the fixed target position for free EDTA has been replaced with the actual position in experimental spectrum *n*
mn(1:57 × 1:57)	The interference matrix, where the contribution from free EDTA has been optimised for the conditions in experimental spectrum *n*
xn (1:57, n)	The reporter signal heights of all compounds in experimental spectrum *n*
c_sample_uM(1:57, n)	The concentrations (μM) of all compounds in NMR sample *n*. Note that the three EDTA compounds were not interpreted quantitatively.
library_xn (1:42000 × 1:57)	The library spectra that corresponds to the reporter signals in experimental spectrum *n* (for proof-of-concept figures only; red)
library_xn_sum(1:42000, n)	Sum line of all library spectra that corresponds to the reporter signals in experimental spectrum *n* (for proof-of-concept figures only; blue)

[a] Note that the input data required for a non-improved and an improved AQuA does not differ

[b] The process to create the (fixed) compound library, do spectral binning in the ChenomX software and import a data file with binning results to MATLAB has been described previously in Röhnisch, H. E.; Eriksson, J.; Müllner, E.; Agback, P.; Sandström, C.; Moazzami, A. A. *Analytical Chemistry* **2018**, *90*, 2095-2102

[c] Datasets required to test this implementation (e.g., from exp_spectra) can be provided upon reasonable request

Table S4: Additional information data used for the improved AQuA implementation

Nr	Compound	Library concentrations (µM) lib_conc_um	Library peak-picking windows lib_window		Experimental peak-picking windows exp_window	
1	2-Aminobutyric acid	4505.3	34666	34678	34667	34675
2	2-Hydroxybutyric acid	4345.4	35063	35073	35056	35073
3	2-Hydroxyisovaleric acid	4477.0	35358	35368	35358	35368
4	2-Oxoglutaric acid (2-Ketoglutaric acid)	7043.8	24515	24525	24511	24525
5	2-Oxoisocaproic acid	2106.1	34840	34850	34840	34850
6	3-Hydroxybutyric acid	4368.3	33527	33537	33527	33537
7	3-Methyl-2-oxovaleric acid	4420.6	35086	35096	35086	35096
8	Acetic acid	2477.8	29957	29967	29952	29967
9	Acetoacetic acid	3544.3	28157	28167	28155	28167
10	Acetone	1441.0	28388	28398	28388	28398
11	Alanine	4884.8	32184	32194	32184	32194
12	Arginine	7938.9	31219	31229	31223	31235
13	Asparagine	22305.6	25345	25355	25345	25355
14	Betaine	953.6	23235	23245	23235	23255
15	Carnitine	985.9	23427	23437	23427	23435
16	Choline	994.9	23549	23559	23545	23559
17	Citric acid	9524.1	26320	26330	26315	26330
18	Creatine	2154.0	24374	24384	24374	24387
19	Creatinine	2702.3	19255	19265	19250	19265
20	Dimethyl sulfone ($DMSO_2$)	1093.1	23790	23800	23789	23799
21	EDTA (free)	-	21370	21390	21360	21415
22	EDTA (calcium)	-	23830	23860	23830	23860
23	EDTA (magnesium)	-	26035	26055	26030	26075
24	Ethanol	5452.5	33633	33643	33633	33643
25	Formic acid	9393.4	279	289	272	298
26	Glucose	22217.8	20004	20014	20000	20014
27	Glutamic acid	20097.3	27769	27779	27775	27782
28	Glutamine	15838.3	27180	27190	27180	27195
29	Glycerol	9839.8	21655	21665	21655	21668
30	Glycine	3246.3	21738	21748	21738	21748
31	Hippuric acid	8233.5	3345	3360	3335	3380
32	Histidine	12962.1	7000	7050	7028	7052
33	Isoleucine	4935.3	34479	34489	34479	34489
34	Isopropanol (2-Propanol)	2064.1	33660	33670	33658	33670
35	Lactic acid	3906.7	32938	32948	32938	32948
36	Leucine	3743.7	34695	34705	34695	34712
37	Lysine	10589.0	24411	24421	24411	24425
38	Methanol	2511.3	22747	22757	22747	22757
39	Methionine	2155.9	26336	26346	26344	26355
40	Methylguanidine	2871.8	25415	25425	25413	25423
41	myo-Inositol	8191.7	19210	19220	19210	19222
42	O-Acetylcarnitine (Acetylcarnitine)	960.3	23590	23600	23590	23600
43	Ornithine	7729.1	24272	24282	24272	24285
44	Phenylalanine	12378.1	5933	5943	5933	5949
45	Proline	25010.6	18827	18837	18827	18837
46	Propionic acid	4835.3	34273	34283	34273	34283
47	Propylene glycol (1,2-propanediol)	3947.8	33815	33825	33813	33830
48	Pyruvic acid	2901.2	27683	27693	27683	27693
49	Sarcosine	2007.9	25860	25870	25863	25873
50	Serine	16985.8	19780	19790	19775	19790
51	Succinic acid	1503.6	27537	27547	27535	27543
52	Threonine	4712.5	18284	18294	18284	18294
53	Trigonelline	3595.5	17355	17365	17355	17365
54	Trimethylamine	867.6	25059	25069	25059	25069
55	Trimethylamine-N-oxide (TMAO)	671.0	23220	23225	23217	23224
56	Tyrosine	10965.2	6550	6560	6547	6565
57	Valine	4976.4	34379	34389	34379	34389

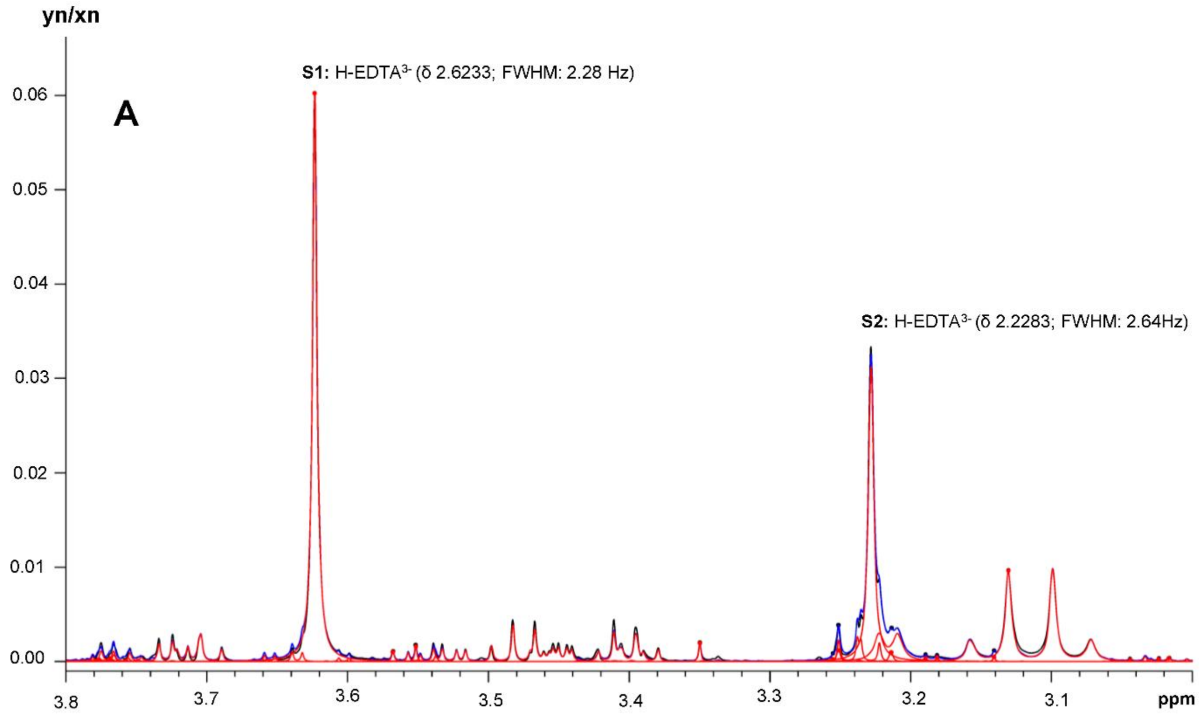

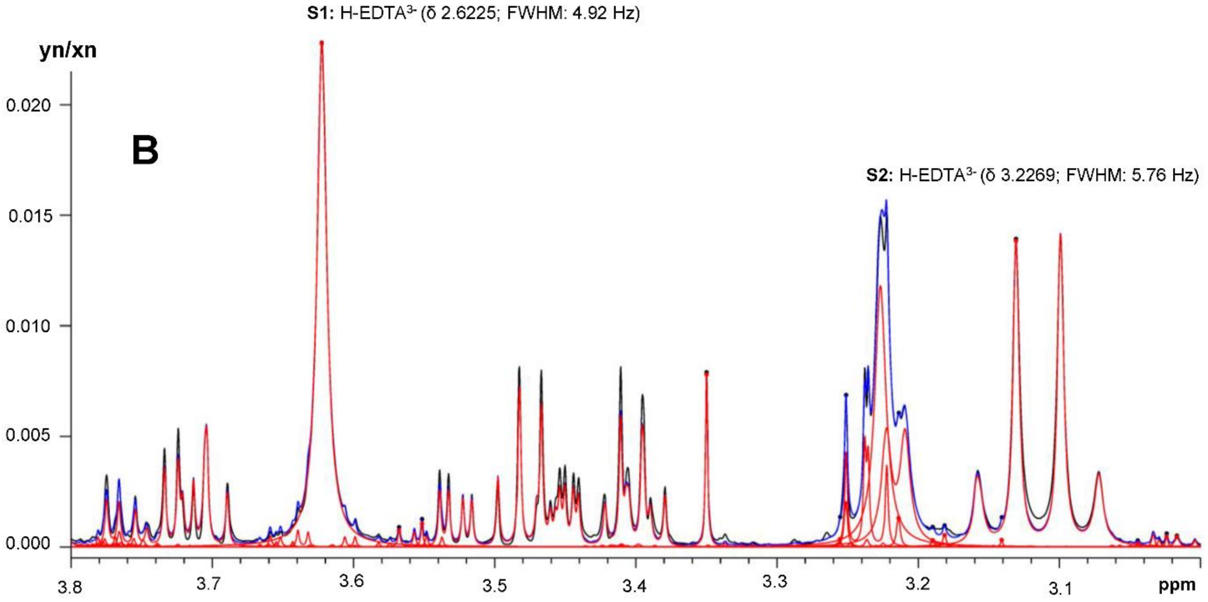

Figure S3: Proof-of concept figures for the improved AQuA implemented in MATLAB. (A) Results for an experimental spectrum with narrow, high-intensity free EDTA signals (n=1). (B) Results for an experimental spectrum with broader, low-intensity free EDTA signals, slightly shifted up-field (n≠1). Data from the improved AQuA: black dots, yn; red dots, xn. Proof-of-concept spectral lines: black, exp_spectra; library_xn, red; library_xn_sum, blue.

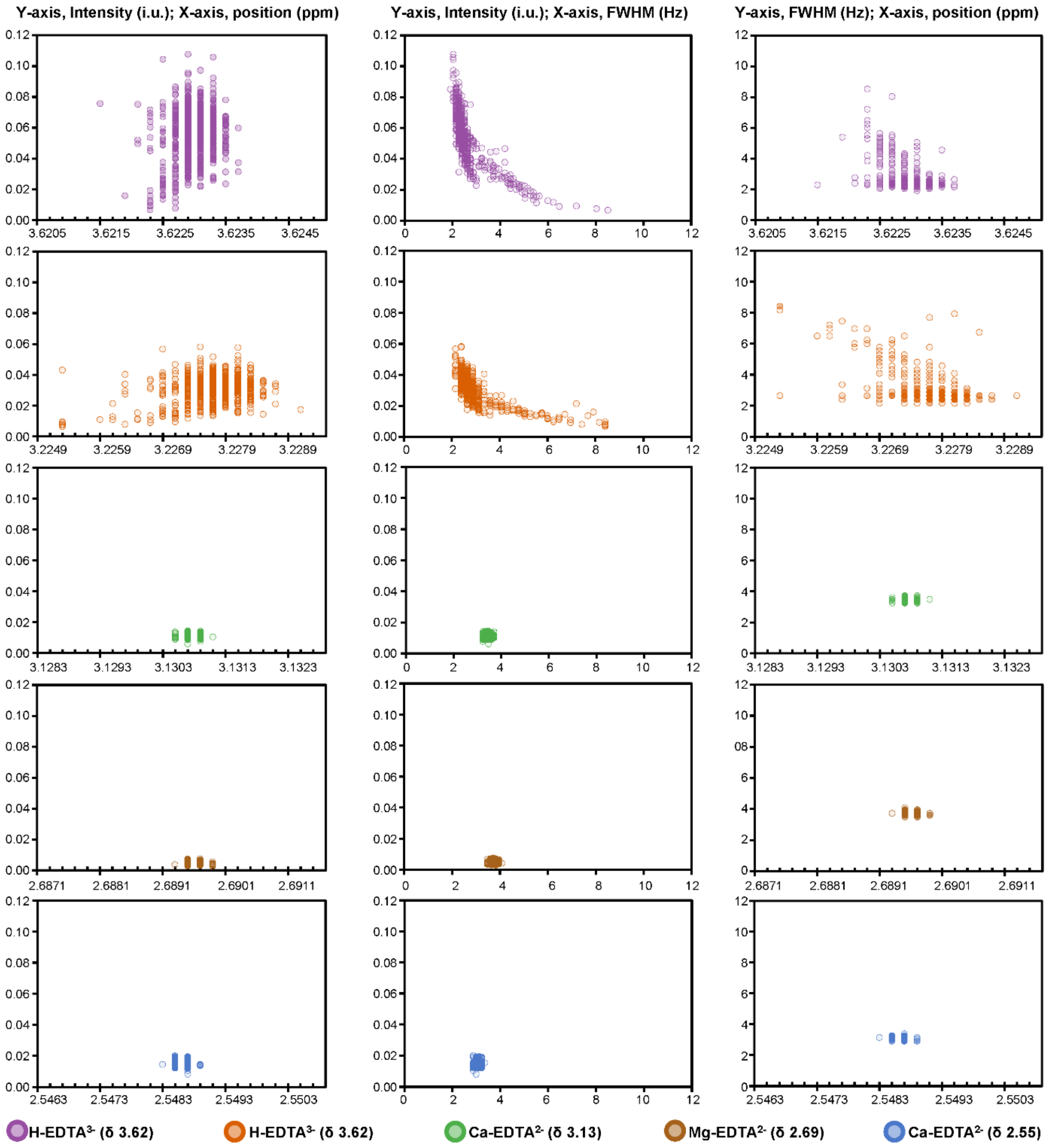

Figure S4: Intensities, positions and line widths of different EDTA signals (n=772). Left panel, intensity (i.u.) versus position (ppm); middle panel, intensity (i.u.) versus FWHM (Hz); right panel, FWHM (Hz) versus position (ppm). The colour of the circles indicates the identity of the signal: purple, H-EDTA^{3-} at δ 3.63 ppm; orange, H-EDTA^{3-} at δ 3.23 ppm; green, Ca-EDTA^{2-} at δ 3.13 ppm; brown, Mg-EDTA at δ 2.69 ppm; blue, Ca-EDTA^{2-} at δ 2.55 ppm. For the left and right panel, the distance between the minor ticks on the x-axes are 0.0002 ppm (1 bin). **Abbreviations:** FWHM, full width at half-maximum; Hz, hertz; i.u., intensity units; ppm, parts per million.

Table S5: Quality indicators for metabolites quantified with the improved AQuA [a, b, c, d]

Metabolite	Occurrence (%)	δ_y (ppm); PD (± bin)	F_q values (from metabolites)		F_q values (from EDTA)	
			q=0.05	*q=0.50*	*q=0.05*	*q=0.50*
1, 2-Propanediol	49	1.1355; 3	0.093	0.000	0.000	0.000
1-Methylguanidine	23	2.8167; 2	0.000	0.000	0.972	0.000
2-Aminobutyric acid	100	0.9665; 1	1.000	0.439	0.000	0.000
2-Hydroxybutyric acid	100	0.8871; 1	0.035	0.000	0.000	0.000
2-Hydroxyisovaleric acid	12	0.8277; 1	0.000	0.000	0.000	0.000
2-Ketoglutaric acid	95	2.9967; 1	1.000	0.000	1.000	0.000
2-Oxoisocaproic acid	72	0.9307; 1	1.000	0.359	0.000	0.000
2-Propanol	90	1.1677; 1	0.628	0.000	0.000	0.000
3-Hydroxybutyric acid	100	1.1939; 1	0.008	0.000	0.000	0.000
3-Methyl-2-oxovaleric acid	11	0.8819; 2	0.927	0.000	0.000	0.000
Acetic acid	99	1.9083; 1	1.000	0.243	0.000	0.000
Acetoacetic acid	95	2.2687; 1	0.999	0.311	0.000	0.000
Acetone	100	2.2213; 1	0.245	0.000	0.000	0.000
Acetylcarnitine	100	3.1813; 1	0.223	0.000	1.000	0.004
Alanine	100	1.4619; 1	0.000	0.000	0.000	0.000
Arginine	70	1.6545; 3	1.000	0.390	0.000	0.000
Asparagine	10	2.8305; 2	0.000	0.000	1.000	0.000
Betaine	100	3.2511; 1	1.000	1.000	0.969	0.000
Carnitine	99	3.2139; 1	0.005	0.000	1.000	0.999
Choline	100	3.1895; 1	0.070	0.000	1.000	0.376
Citric acid	100	2.6357; 1	0.000	0.000	0.313	0.000
Creatine	100	3.0239; 1	0.997	0.000	0.889	0.000
Creatinine	100	4.0487; 1	0.614	0.000	0.000	0.000
$DMSO_2$	97	3.1407; 2	0.000	0.000	1.000	0.996
Ethanol	96	1.1727; 1	0.843	0.000	0.000	0.000
Formic acid	70	8.4427; 1	0.000	0.000	0.000	0.000
Glucose	100	3.8989; 1	0.000	0.000	0.000	0.000
Glutamic acid	57	2.3441; 1	1.000	0.545	0.000	0.000
Glutamine	100	2.4625; 1	0.000	0.000	0.051	0.000
Glycerol	100	3.5677; 1	0.021	0.000	0.930	0.000
Glycine	100	3.5515; 1	0.825	0.000	0.019	0.000
Histidine	100	7.0929; 4	0.000	0.000	0.000	0.000
Isoleucine	100	1.0033; 1	0.026	0.000	0.000	0.000
Lactic acid	100	1.3117; 1	0.723	0.000	0.000	0.000
Leucine	100	0.9595; 1	1.000	0.000	0.000	0.000
Lysine	100	3.0165; 1	0.014	0.000	0.983	0.000

Table S5: (Continued)

Metabolite	Occurrence (%)	δ_y (ppm); PD (± bin)	F_q values (from metabolites)		F_q values (from EDTA)	
			q=0.05	*q=0.50*	*q=0.05*	*q=0.50*
Methanol	100	3.3499; 1	0.171	0.000	0.000	0.000
Methionine	95	2.6311; 1	1.000	0.004	1.000	0.000
Myo-inositol	38	4.0567; 1	1.000	0.000	0.003	0.000
Ornithine	100	3.0445; 1	0.930	0.000	1.000	0.000
Phenylalanine	100	7.3117; 3	0.000	0.000	0.000	0.000
Proline	99	4.1337; 1	0.984	0.001	0.000	0.000
Pyruvic acid	100	2.3629; 1	0.000	0.000	0.000	0.000
Sarcosine	18	2.7265; 1	0.000	0.000	1.000	0.000
Serine	100	3.9439; 1	1.000	0.000	0.000	0.000
Succinic acid	100	2.3927; 2	1.000	0.131	0.000	0.000
Threonine	98	4.2427; 1	0.000	0.000	0.000	0.000
TMAO	100	3.2553; 0	1.000	0.049	0.999	0.000
Trimethylamine	46	2.8871; 2	0.000	0.000	0.997	0.000
Tyrosine	100	7.1889; 1	0.000	0.000	0.000	0.000
Valine	100	1.0233; 1	0.000	0.000	0.000	0.000

[a] Hippuric acid, propionic acid and trigonelline displayed an *occurrence* < 5% and where therefore not investigated further

[b] Values below the detection limit were excluded, except when computing the *occurrences*

[c] Histidine displayed lower *positional deviation* in the present study compared to our previous study (Röhnisch, H. E.; Eriksson, J.; Müllner, E.; Agback, P.; Sandström, C.; Moazzami, A. A. *Analytical Chemistry* **2018**, *90*, 2095-2102) where heparin was used as anticoagulant. EDTA can reduce ionic strength dependent positional deviations of signals (Asiago, V. M.; Nagana Gowda, G. A.; Zhang, S.; Shanaiah, N.; Clark, J.; Raftery, D. *Metabolomics* **2008**, *4*, 328-336)

[d] The target signals were selected outside the water signal region. Water suppression (done to avoid dynamic range issues caused by the larger water content in the samples) can attenuate metabolite signals located in proximity to the water region (Hwang, T-L.; Shaka, A. *Journal of Magnetic Resonance, Series A*. **1995**, *112*, 275-279).

Abbreviations: $DMSO_2$, dimethylsulfone; PD, positional deviation, ppm, parts per million; TMAO, trimethylamine-N-oxide

Table S6: Separating the interference into different sources [a, b]

Compound Nr/Source	Target position	Variable of interest	Total contribution	Separation into individual contributions from: Reporter	Source A	Source B
1/A	δ_1	Target height y_1	$\Sigma_{i=1}^{5}\ (m_{1,i} \cdot x_i)$	$x_1\ (m_{1,1} \cdot x_1;\ m_{1,1}=1)$	$m_{1,2} \cdot x_2$	$m_{1,3} \cdot x_3 + m_{1,4} \cdot x_4 + m_{1,5} \cdot x_5$
		Interference Δ_1	$(y_1 - x_1)/y_1$	NA	$(m_{1,2} \cdot x_2)/y_1$	$(m_{1,3} \cdot x_3 + m_{1,4} \cdot x_4 + m_{1,5} \cdot x_5)/y_1$
2/A	δ_2	Target height y_2	$\Sigma_{i=1}^{5}\ (m_{2,i} \cdot x_i)$	$x_2\ (m_{2,2} \cdot x_2;\ m_{2,2}=1)$	$m_{2,1} \cdot x_1$	$m_{2,3} \cdot x_3 + m_{2,4} \cdot x_4 + m_{2,5} \cdot x_5$
		Interference Δ_2	$(y_2 - x_2)/y_2$	NA	$(m_{2,1} \cdot x_1)/y_2$	$(m_{2,3} \cdot x_3 + m_{2,4} \cdot x_4 + m_{2,5} \cdot x_5)/y_2$
3/B	δ_3	Target height y_3	$\Sigma_{i=1}^{5}\ (m_{3,i} \cdot x_i)$	$x_3\ (m_{3,3} \cdot x_3;\ m_{3,3}=1)$	$m_{3,1} \cdot x_1 + m_{3,2} \cdot x_2$	$m_{3,4} \cdot x_4 + m_{3,5} \cdot x_5$
		Interference Δ_3	$(y_3 - x_3)/y_3$	NA	$(m_{3,1} \cdot x_1 + m_{3,2} \cdot x_2)/y_3$	$(m_{3,4} \cdot x_4 + m_{3,5} \cdot x_5)/y_3$
4/B	δ_4	Target height y_4	$\Sigma_{i=1}^{5}\ (m_{4,i} \cdot x_i)$	$x_4\ (m_{4,4} \cdot x_4;\ m_{4,4}=1)$	$m_{4,1} \cdot x_1 + m_{4,2} \cdot x_2$	$m_{4,3} \cdot x_3 + m_{4,5} \cdot x_5$
		Interference Δ_4	$(y_4 - x_4)/y_4$	NA	$(m_{4,1} \cdot x_1 + m_{4,2} \cdot x_2)/y_4$	$(m_{4,3} \cdot x_3 + m_{4,5} \cdot x_5)/y_4$
5/B	δ_5	Target height y_5	$\Sigma_{i=1}^{5}\ (m_{5,i} \cdot x_i)$	$x_5\ (m_{5,5} \cdot x_5;\ m_{5,5}=1)$	$m_{5,1} \cdot x_1 + m_{5,2} \cdot x_2$	$m_{5,3} \cdot x_3 + m_{5,4} \cdot x_4$
		Interference Δ_5	$(y_5 - x_5)/y_5$	NA	$(m_{5,1} \cdot x_1 + m_{5,2} \cdot x_2)/y_5$	$(m_{5,3} \cdot x_3 + m_{5,4} \cdot x_4)/y_5$

[a] Modified AQuA computation for experimental spectrum *n* ($\bar{\mathbf{y}}_\mathbf{n} = \bar{\bar{\mathbf{m}}}_\mathbf{n} \cdot \bar{\mathbf{x}}_\mathbf{n}$) exemplified for *5* compounds:

$y_1 = m_{1,1} \cdot x_1 + m_{1,2} \cdot x_2 + m_{1,3} \cdot x_3 + m_{1,4} \cdot x_4 + m_{1,5} \cdot x_5$
$y_2 = m_{2,1} \cdot x_1 + m_{2,2} \cdot x_2 + m_{2,3} \cdot x_3 + m_{2,4} \cdot x_4 + m_{2,5} \cdot x_5$
$y_3 = m_{3,1} \cdot x_1 + m_{3,2} \cdot x_2 + m_{3,3} \cdot x_3 + m_{3,4} \cdot x_4 + m_{3,5} \cdot x_5$
$y_4 = m_{4,1} \cdot x_1 + m_{4,2} \cdot x_2 + m_{4,3} \cdot x_3 + m_{4,4} \cdot x_4 + m_{4,5} \cdot x_5$
$y_5 = m_{5,1} \cdot x_1 + m_{5,2} \cdot x_2 + m_{5,3} \cdot x_3 + m_{5,4} \cdot x_4 + m_{5,5} \cdot x_5$

Interference matrix element $m_{i,j}$: obtained after normalisation of the intensity value extracted from the metabolite library at position δ_i from compound j (≥ 0; if $i = j$, then $m_{i,j} = 1$); Target height y_i: the height of the experimental signal selected for quantification of compound i (modelled as the sum of intensity contributions at position δ_i from: compound i, reporter; compounds $\neq i$, interferences); Reporter x_i: the estimated height of the pure signal from compound i at position δ_i; Interference Δ_i: the sum of relative height contributions to y_i from compounds $\neq i$

[b] In this study, the total interference was separated into two sources:

A) From non-metabolites (EDTA)
B) From metabolites

Abbreviations: EDTA; ethylenediamine-tetra-acetic acid; NA, not applicable (since the reporter is not an interference); Nr, number

Table S7: Comparison of mean sample concentrations [a, b, c]

Metabolite	$\mu_{Heparin}$	μ_i	μ_{ii} or μ_{EDTA}	$(\mu_{EDTA}-\mu_{Heparin})/\mu_{Heparin}$	$(\mu_i-\mu_{ii})/\mu_{ii}$
1, 2-Propanediol	1.4	2.6	2.6	0.850	0.003
2-Aminobutyric acid	6.7	5.0	5.0	-0.251	0.001
2-Hydroxybutyric acid	9.6	8.2	8.2	-0.149	0.001
2-Hydroxyisovaleric acid	2.0	1.4	1.4	-0.328	0.004
2-Oxoisocaproic acid	1.7	1.2	1.2	-0.326	0.002
2-Propanol	1.5	3.2	3.2	1.076	0.001
3-Hydroxybutyric acid	19.3	14.4	14.3	-0.255	0.001
3-Methyl-2-oxovaleric acid	2.0	1.6	1.6	-0.242	0.004
Acetic acid	9.8	7.1	7.5	-0.235	-0.054
Acetoacetic acid	6.9	6.1	6.1	-0.125	0.011
Acetone	2.8	2.8	2.8	-0.003	0.007
Alanine	88.2	95.8	95.9	0.088	-0.001
Arginine	30.6	29.7	29.6	-0.035	0.005
Creatinine	19.0	14.7	15.1	-0.207	-0.024
Ethanol	7.2	5.7	5.7	-0.204	0.002
Formic acid	7.4	9.1	9.1	0.235	0.000
Glucose	985.4	1018.9	1018.2	0.033	0.001
Glutamic acid	11.6	10.2	9.8	-0.154	0.047
Histidine	22.8	18.0	18.0	-0.210	0.000
Isoleucine	16.8	16.7	16.7	-0.01	0.000
Lactic acid	382.6	337.2	337.5	-0.118	-0.001
Leucine	30.3	27.1	27.1	-0.106	0.000
Methanol	16.5	69.3	68.7	3.169	0.008
Myo-inositol	7.2	4.2	5.6	-0.225	-0.243
Phenylalanine	11.1	11.2	11.2	0.010	0.001
Proline	49.1	53.3	52.7	0.073	0.010
Pyruvic acid	19.1	21.9	21.8	0.145	0.004
Serine	27.6	27.4	26.9	-0.023	0.019
Succinic acid	0.9	2.1	2.2	1.485	-0.018
Threonine	29.0	27.4	27.1	-0.067	0.014
Tyrosine	16.9	18.8	18.8	0.112	0.000
Valine	62.6	58.9	58.9	-0.058	0.000
Glycine	46.9	57.2	55.9	0.192	0.023
Glutamine	116.9	119.0	115.5	-0.012	0.030
Citric acid	32.0	61.3	58.8	0.838	0.042
Creatine	5.2	10.3	9.5	0.831	0.084

Table S7: (Continued)

**Metabolite**	$\mu_{Heparin}$	μ_i	μ_{ii} and μ_{EDTA}	$(\mu_{EDTA}-\mu_{Heparin})/\mu_{Heparin}$	$(\mu_i-\mu_{ii})/\mu_{ii}$
Glycerol	284.3	95.8	88.2	-0.690	0.086
1-Methylguanidine	0.7	1.3	1.0	0.417	0.205
Betaine	14.0	16.5	13.3	-0.054	0.246
Lysine	42.8	48.7	45.3	0.058	0.075
2-Ketoglutaric acid	3.7	6.0	4.4	0.185	0.348
Acetylcarnitine	2.2	5.9	3.7	0.675	0.603
Asparagine	13.4	16.2	13.1	-0.020	0.236
Methionine	8.0	8.3	6.3	-0.212	0.324
Ornithine	13.7	19.2	12.8	-0.065	0.497
Sarcosine	0.5	1.1	0.5	-0.082	1.427
Trimethylamine	0.5	0.5	0.4	-0.203	0.182
TMAO	2.8	4.1	2.5	-0.109	0.658
Choline	3.0	6.3	3.2	0.051	0.996
Carnitine	11.4	37.1	10.7	-0.068	2.483
$DMSO_2$	2.5	10.4	2.3	-0.057	3.412

[a] Explanation of table columns:

$\mu_{heparin}$: mean sample concentrations derived with the non-modified AQuA implemented for dataset heparin (n=1342). Note that these results were derived previously by Röhnisch, H. E.; Eriksson, J.; Müllner, E.; Agback, P.; Sandström, C.; Moazzami, A. A. *Analytical Chemistry* **2018**, *90*, 2095-2102; μ_i: mean sample concentrations derived with the non-modified AQuA implemented for dataset EDTA (n=772); μ_i and μ_{EDTA}: mean sample concentrations derived with the non-modified AQuA implemented for dataset EDTA (n=772); $(\mu_{EDTA} - \mu_{Heparin})/\mu_{Heparin}$: Comparison of mean concentrations derived by the non-modified AQuA between dataset EDTA and dataset Heparin; $(\mu_i - \mu_{ii})/\mu_{ii}$: Comparison of mean concentrations derived for dataset EDTA between the results from the non-modified and the modified AQuA

[b] Metabolites are sorted by F_q values (from EDTA)

[c] Note that the mean concentrations (μM) corresponds to the concentrations in the NMR sample and final plasma concentrations can be derived by accounting for the dilution (dilution factor: 4.25)

Abbreviations: AQuA, automated quantification algorithm; $DMSO_2$, dimethyl sulfone; EDTA, ethylenediamine-tetra-acetic acid; TMAO, trimethylamine-N-oxide

Table S8: Workflow for comparing the improved AQuA to different ASICS approaches [a, b, c]

	**Data tested with ASICS**
**Experimental (N=30)**	A subset of 30 experimental spectra (binned data normalised based on the TSP signal area), which had already been quantified with the improved AQuA, were randomly selected from the entire dataset for quantification with ASICS.
**Simulated[a] (N=30+30)**	Simulated spectra representing known mixtures of metabolite concentrations (μM), were generated by summing normalised library spectra weighed by their respective reporter contributions in the improved AQuA computations. Simulated spectrum *n* used $\bar{\mathbf{x}}_{\mathbf{n}}$ vector elements resulting from the AQuA computation on experimental spectrum *n*. Two subsets of simulated metabolite spectra were generated for quantification with ASICS: one subset contained 30 spectra including 54 metabolites and 3 EDTA compounds and the other subset contained 30 spectra with only the metabolites.
	**Libraries used in ASICS**
**Library spectra (N=56+30+1)**	ASICS allows the user to choose between different approaches for reference library alignment and metabolite quantification (R, G, B, Y, see below), and also to import reference libraries. To facilitate a straightforward comparison between the improved AQuA and ASICS, it was desirable to use the same compound library in both algorithms (i.e., the library used in the improved AQuA). For all compounds (except free EDTA) the fixed library spectra used in the improved AQuA could also be utilised in ASICS (N=56). When applicable, the free EDTA library spectrum generated in the nth improved AQuA computation was used for the nth ASICS computation (*n*=1:30). This could be applied to one of the ASICS approaches described below (R**). In the other ASICS approaches the average free EDTA library spectrum was used (N=1).
	**Preparation for ASICS**
**Simulated/ Experimental**	Before importing the experimental- and simulated datasets into RStudio, a synthetic signal (Lorentzian: position, 0 ppm; intensity, 1; line width 1.15 Hz) was added to each spectrum. By using the synthetic signal and the PepsNMR peak method included in the ASICS package, the mandatory normalisation step in ASICS could be performed without changing the spectra. Data was scaled by a factor of 10^6 to minimise the amount of zero value ASICS quantifications. No alignment was done prior to quantification.
**Libraries**	The library spectra were normalised by a constant sum. The number of protons (nb.protons) for each compound was set according to its molecular formula without amino- and hydroxyl protons and the threshold was set to 0.1 to obtain a smooth transition between signals and baseline.
	**ASICS approaches tested**
**Red (R/R**)**	Independent library alignment and independent quantification with FWER-controlled compound selection
**Blue (B)**	Joint library alignment, independent quantification, and FWER-controlled compound selection
**Green (G)**	Joint library alignment and independent quantification followed by FWER-controlled compound selection for final joint quantification
**Yellow (Y)**	Joint library alignment and joint quantification
	**ASICS parameters used**
**Max.shift**	Library signals were allowed to deviate 0.01 ppm (max.shift=0.01; 50 bins), a number that exceeds the *positional deviations* typically observed in the entire dataset. Other parameters were kept at their default values.

[a] The known metabolite concentrations were simulated to reflect the experimental dynamic range and free EDTA signals were simulated to reflect the experimentally observed inter-spectral positional and line width variations

[b] ASICS package (Version: 2.6.1) git_url: https://git.bioconductor.org/packages/ASICS

[c] The ASICS package was employed in Rstudio (version 4.0.4); R Core Team (2021). R: A language and environment for statistical computing. R Foundation for Statistical Computing, Vienna, Austria. URL https://www.R-project.org/)

References: Tardivel, P.; Canlet, C.; Lefort, G.; Tremblay-Franco, M.; Debrauwer, L.; Concordet, D.; Servien, R. *Metabolomics* **2017**, *13*, 109; Lefort, G.; Liaubet, L.; Canlet, C.; Tardivel, P.; Père, M.C.; Quesnel, H.; Paris, A.; Iannuccelli, N.; Vialaneix, N.; Servien R. *Bioinformatics* **2019**, *35*, 4356–4363; Lefort, G.; Liaubet, L.; Marty-Gasset, N.; Canlet, C.; Vialaneix, N.; Servien, R. *Analytical Chemistry* **2021**, *93*, 2861-2870.

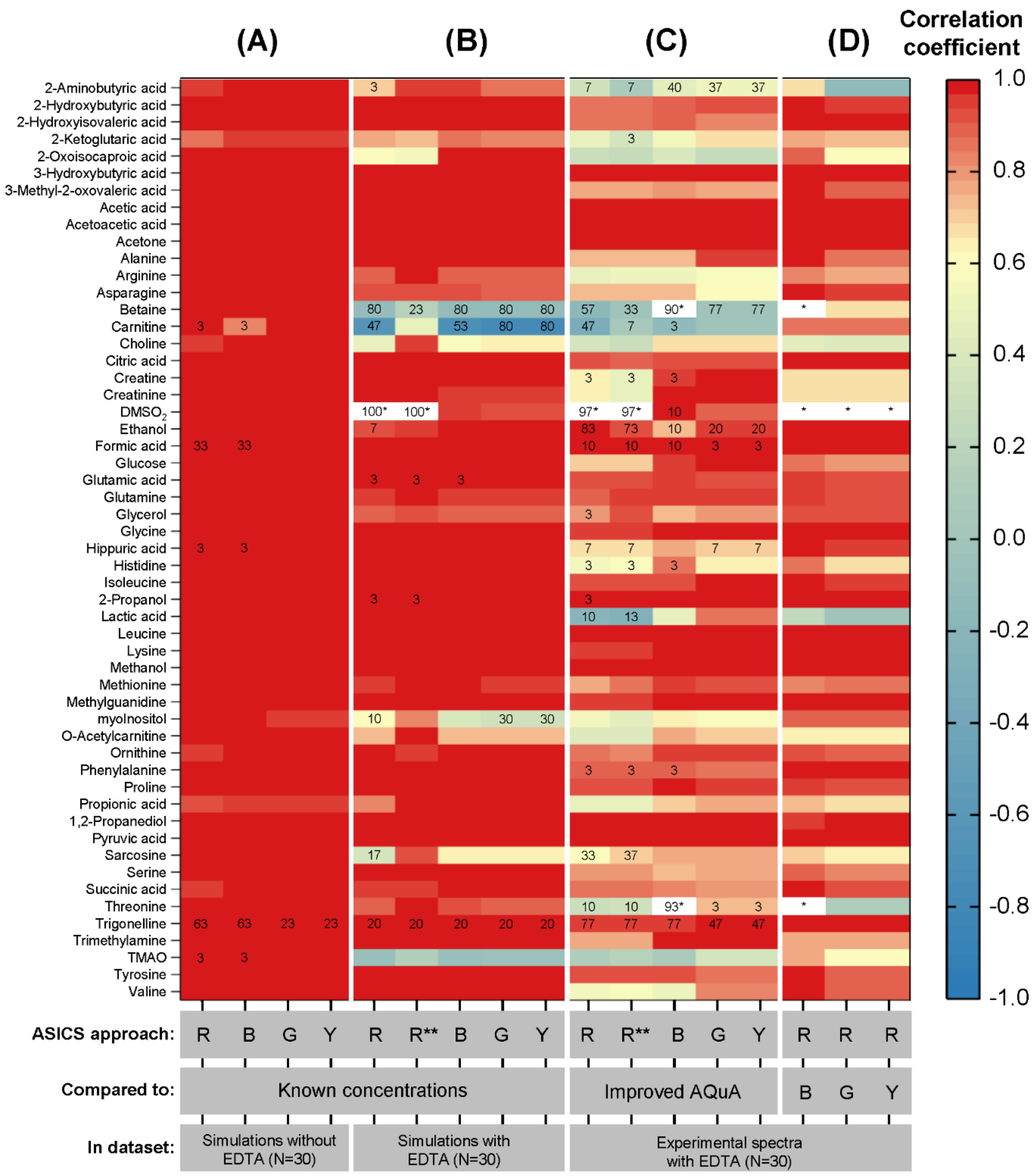

Figure S5: Correlation heatmap for 54 metabolites. (A) Known concentrations, µM, compared to estimated relative concentration estimates from ASICS approaches in simulations without EDTA (N=30). (B) Known concentrations compared the ASICS approaches in simulations with EDTA (N=30). (C) Concentration estimates from the improved AQuA, µM), compared to ASICS approaches in experimental spectra with EDTA (N=30). (D) Estimates from the independent ASICS approach (R) compared to the joint ASICS approaches (B, R and Y) in experimental spectra with EDTA (N=30). ASICS approaches: "R", independent library alignment and independent quantification with FWER-controlled compound selection; "B", joint library alignment, independent quantification, and FWER-controlled compound selection; "G", joint library alignment and independent quantification followed by FWER-controlled compound selection for joint quantification; "Y", joint library alignment and joint quantification. *ASICS zero values (%) are shown in black inside the colored fields of the figure and no correlation coefficient is displayed if the missing data >90%. The independent approach (R**) was performed with the nth library spectrum for free EDTA (generated in the improved AQuA) for the nth ASICS computation. In all other approaches the average free EDTA library spectrum was used.

Table S9: General observations and interpretations of comparisons with ASICS

General observation from Figure S5	Interpretation
●**(A):** The concentrations estimates derived with all four ASICS approaches (R, B, G, and Y) were typically highly correlated to known concentration in simulations without EDTA	● The employed ASICS processing workflow (e.g., normalisation, scaling and compound library) performed adequately as the results correlated well with the known values
●**(A-B):** Correlations between ASICS and known concentrations were typically higher in simulations without EDTA signals compared to simulations with EDTA	● In the presence of EDTA signals, quantification with ASICS can be more difficult
●**(B):** In simulations with EDTA, the independent procedure that accounted for inter-spectral line width variations of free EDTA (R**) showed somewhat higher correlations to known concentrations than the independent procedure that did not adjust for such line width variations (R)	● By utilising library data generated by the improved AQuA in the independent ASICS procedure (R**) it becomes possible to account for inter-spectral line width variation of free EDTA signals to possibly improve the outcome. (We were not able to find a straightforward way to account for such line width variations with the joint procedures (B, G and Y), since these procedures use one library for an entire dataset)
●**(C):** In experimental spectra with EDTA, correlations between the improved AQuA and ASICS were typically higher for the joint procedures (B, G, Y) compared to the independent procedures (R, R**)	● Overall, the improved AQuA yielded results more similar to the joint ASICS approaches
●**(D):** In experimental spectra with EDTA, some metabolites showed low correlations between the independent (R) and joint (B, G, Y) ASICS approaches	● When the different ASICS approaches were employed on the same experimental dataset, with the same library and parameter settings, the outcome differed for some metabolites. (some joint procedures (G, Y) yields extremely similar outcome)
